# Temporal multi-omic exploration of the ventral tegmental area in chronic pain and passive coping behaviors

**DOI:** 10.1016/j.isci.2026.116085

**Published:** 2026-05-22

**Authors:** Cody C. Diezel, Lisa A. Majuta, Erfan Bahramnejad, Kelly L. Karlage, Jennifer L. Partin, Saniya M.D. Barbour, Ingrid L. Peterson, Matthew Flowers, Sophia T. von Hippel, Riley Haveman, Ethan Villarroel, Isabella Villarroel, Paul R. Langlais, Tally M. Largent-Milnes, Todd W. Vanderah, Arthur C. Riegel

**Affiliations:** 1 Comprehensive Center for Pain and Addiction (CCPA), University of Arizona, Tucson, AZ 85724, USA; 2 Department of Pharmacology, College of Medicine, University of Arizona, Tucson, AZ 85724, USA; 3 Department of Medicine, Division of Endocrinology, University of Arizona, Tucson, AZ 85724, USA; 4 Department of Neuroscience, College of Science, University of Arizona, Tucson, AZ 85724, USA; 5 James C. Wyant College of Optical Sciences, University of Arizona, Tucson, AZ 85724, USA

**Keywords:** molecular neuroscience, cell, proteomics

## Abstract

The comorbidity of chronic pain and mood disorders poses a clinical challenge involving neurobiological mechanisms within key brain regions like the ventral tegmental area (VTA). We employed a multidisciplinary approach in male mice, combining proteomics, phosphoproteomics, and lipidomics with behavioral and immunohistochemical analyses in a neuropathic pain model. Our findings reveal a temporal evolution of the VTA molecular landscape—an early signature related to metabolic reallocation followed by a late maladaptive state characterized by shifts in energy metabolism and cytoskeletal remodeling. This late maladaptive state involved stoichiometric remodeling of Kv7 potassium channel subunits and depletion of the endocannabinoid 2-arachidonoylglycerol (2-AG), coinciding with the passive coping behavior. Pharmacological administration of 2-AG or potentiation of Kv7 channel function reversed pain-induced passive coping. Together, these findings delineate a temporal molecular evolution in the VTA and validate 2-AG and Kv7 systems as regulatory nodes linking chronic pain to the emergence of passive coping behaviors.

## Highlights

• Neuropathic pain shifts the VTA from early adaptation to late-stage cellular stress
• Chronic pain depletes VTA 2-AG, removing a critical presynaptic inhibitory brake
• Chronic pain remodels VTA Kv7 in dopamine neurons, weakening postsynaptic M-current brakes
• Restoring 2-AG or Kv7 function reverses pain-induced passive coping

## Graphical abstract

**Figure:**
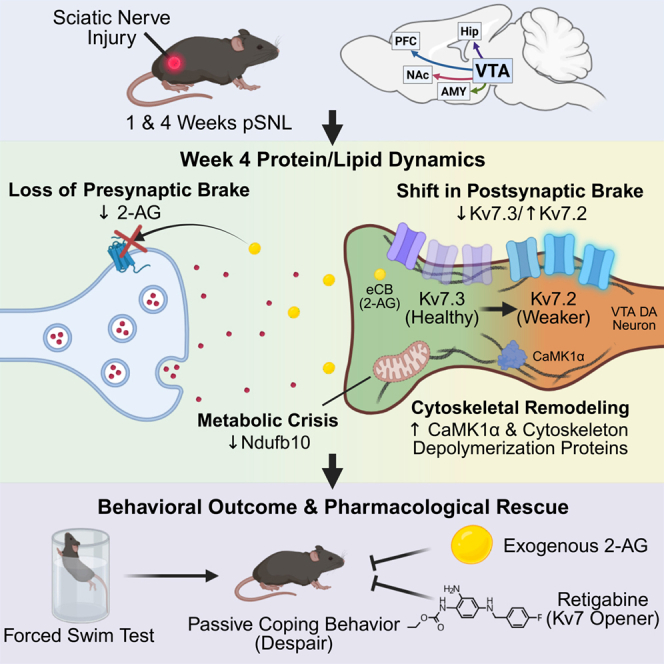

## Introduction

Chronic pain, defined as persistent discomfort lasting three months or longer,^1^ is a global health burden affecting over 20% of adults worldwide and profoundly diminishing quality of life.^2^^,^^3^ Its clinical impact is significantly exacerbated by a high comorbidity with mood disorders; nearly 40%–60% of chronic pain patients experience clinically significant symptoms of major depressive or anxiety disorders.^2^^,^^3^ This bidirectional relationship creates a deleterious cycle where persistent pain drives mood dysregulation, and mood disorders heighten pain perception. Such complexity complicates treatment and underscores the urgent need to unravel the shared neurobiological mechanisms that link these conditions.

The ventral tegmental area (VTA), a central hub in the mesolimbic dopamine system, is increasingly being recognized not merely as a reward center but also as a critical node for integrating pain, motivation, and affective state.^4^ The VTA receives nociceptive input from the ascending pain pathways and projects to the limbic and cortical areas governing emotion, motivation, and decision-making,^4^^,^^5^ which positions it as a translator of nociceptive signals into related affective consequences. Chronic pain drives shifts in the VTA dopamine neuron activity, which correlates with maladaptive behavioral and emotional outcomes.^6^^,^^7^ Thus, characterizing the molecular mechanisms underlying VTA dysfunction in chronic pain is essential for developing treatments that address both the sensory and affective components of pain.

Despite the established role of the VTA in pain-induced mood changes, its specific molecular landscape remains poorly defined. Although previous studies have reported conflicting findings on VTA dopamine neuron excitability in models of depression and pain,^5^^,^^6^^,^^7^^,^^8^^,^^9^^,^^10^^,^^11^ how VTA activity changes during the transition from acute to chronic pain are not well understood. Defining the coordinated molecular remodeling of the VTA—including changes in protein abundance, phosphorylation state, and endocannabinoid (eCB) lipid availability—is necessary to determine which molecular adaptations may underlie the pain-mood comorbidity.

One critical system governing neuronal inhibitory control is the eCB system, a lipid signaling network essential for the homeostasis of neural function. The eCB system primarily comprises two primary eCBs—anandamide (AEA) and 2-arachidonoylglycerol (2-AG)—along with their associated CB1 and CB2 receptors and synthesizing/degradative enzymes.^12^^,^^13^ 2-AG functions as a retrograde messenger and is synthesized postsynaptically by diacylglycerol lipase (DAGL) in response to calcium influx; it travels to bind presynaptic CB1 receptors and suppresses both glutamatergic and GABAergic neurotransmitter release.^14^^,^^15^ This activity-dependent inhibitory brake is essential for maintaining excitatory/inhibitory balance in neural circuits governing pain and mood. Preclinical and clinical evidence demonstrates that eCB signaling is compromised in both chronic pain and mood disorders, where peripheral measures of eCB dysregulation have been correlated with both pain intensity and depressive symptoms in human studies.^16^^,^^17^^,^^18^ Furthermore, pharmacological restoration of peripheral eCB signaling can reverse both pain-like and depression-like behaviors in preclinical models.^19^^,^^20^^,^^21^ However, whether 2-AG levels are specifically altered within the VTA during chronic neuropathic pain, and whether eCB dysregulation in this region causally contributes to affective symptoms, remain unknown.

A second critical system regulating homeostatic excitability is the Kv7 (KCNQ) family of voltage-gated potassium channels, which generate the M current—an outward current that stabilizes the resting membrane potential and regulates repetitive neuronal firing.^22^^,^^23^^,^^24^ Among Kv7 subunits, Kv7.2 and Kv7.3 are predominant in the pain-processing circuits.^25^^,^^26^ These subunits preferentially co-assemble into heteromers that generate a more voltage-sensitive current than Kv7.2 homomers alone.^22^^,^^23^^,^^27^ Kv7 channel composition is dynamic and can be remodeled by metabolic state, calcium signaling, lipid availability (PIP_2_), and cellular stress.^28^^,^^29^^,^^30^ While Kv7 channel dysfunction is well established in peripheral neuropathic pain, where a reduced M current drives sensory neuron firing, evidence also suggests that Kv7 dysfunction may contribute to depression-like behaviors.^31^ Furthermore, Kv7 channel openers can modulate dopamine signaling to produce antidepressant-like effects in behavioral models.^9^^,^^32^ Complementing these behavioral findings, intra-VTA retigabine has been shown to directly modulate VTA neuron electrophysiological properties in neuropathic pain models.^6^^,^^7^ These observations establish Kv7 channels as potential therapeutic targets in pain-mood comorbidity, yet their specific alterations within the VTA during chronic pain and their definitive role in dopamine neuron function have not been fully characterized.

Chronic pain triggers metabolic remodeling in brain regions, driving shifts from oxidative phosphorylation toward autophagy and mitochondrial dysfunction.^33^^,^^34^ These metabolic adaptations may have direct functional consequences because both eCB biosynthesis—which is calcium and ATP dependent—and ion channel trafficking/stability—which requires intact mitochondrial function and cytoskeletal transport—are fundamentally vulnerable to energy failure.^14^^,^^29^ This suggests a mechanistic link wherein metabolic dysregulation in chronic pain could simultaneously compromise multiple inhibitory systems regulating neuronal excitability. Notably, 2-AG and Kv7 channels are functionally complementary: 2-AG suppresses presynaptic neurotransmitter release via CB1 receptors (acting as a presynaptic brake),^13^^,^^14^^,^^15^ while Kv7 M current stabilizes postsynaptic membrane potential and limits spike initiation (acting as a postsynaptic brake).^22^^,^^23^^,^^24^^,^^28^ Convergent dysregulation of both systems is more likely to drive a persistent neuronal excitability shift than dysregulation of either alone.

This study tests the hypothesis that the transition to persistent pain is marked by a VTA shift from an initial adaptive response to a late-stage maladaptive state defined by cellular stress and the convergent loss of 2-AG and Kv7 homeostatic control. To test this, we coupled the partial sciatic nerve ligation (pSNL) model with integrated multi-omic analysis at one and four weeks post-injury, followed by targeted behavioral and pharmacological validation. We used unbiased VTA proteomics to identify molecular signatures at two stages of this putative transition, targeted eCB lipids using lipidomic analysis to determine whether VTA eCB (2-AG or AEA) signaling is altered in chronic pain, and validated key proteomic findings and Kv7 ion channels with immunohistochemistry (IHC) in relation to tyrosine hydroxylase-positive (TH^+^) neurons.

## Results

All experimental statistics evaluated through GraphPad Prism are presented in Table S1.

### pSNL induces persistent neuropathic pain and a delayed shift toward passive coping

**Figure 1:**
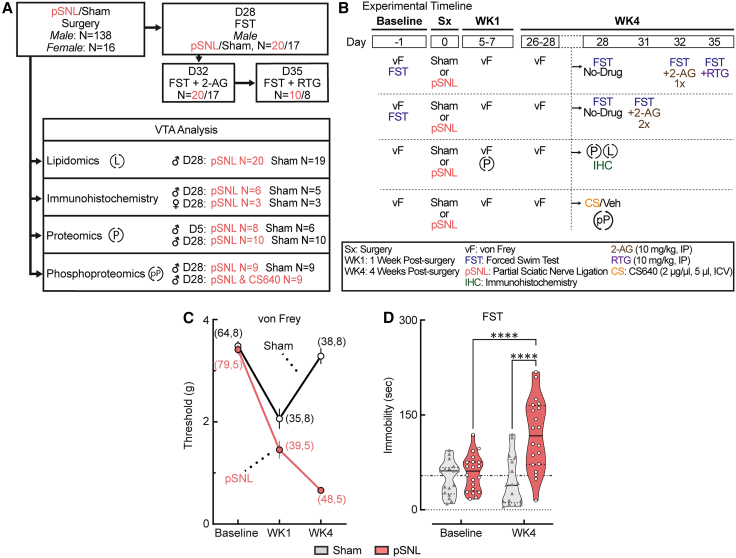
pSNL induces persistent allodynia and delayed passive coping behavior (A and B) Experimental timeline and group allocations for behavioral and molecular analyses at week 1 (WK1) and week 4 (WK4) post-surgery. Behavioral tests included the von Frey (vF) test for mechanical sensitivity and the forced swim test (FST) for coping behavior. Pharmacological interventions prior to the FST included 2-AG (10 mg/kg, i.p.) and retigabine (RTG; 10 mg/kg, i.p.). (C) Paw withdrawal thresholds measured at baseline, WK1, and WK4 post-surgery in male and female mice. A two-way mixed-effects ANOVA revealed significant main effects of time, surgery, and a significant interaction (F [2,182] = 64.77, ∗∗∗∗*p* < 0.0001). Subsequent Tukey post-hoc analysis showed that both pSNL and sham groups exhibited hypersensitivity at WK1 compared to baseline (∗∗∗∗*p* < 0.0001). Hypersensitivity persisted in pSNL mice at WK4 (∗∗∗∗*p* < 0.0001) but resolved in sham mice (*p* = 0.3197). Sample sizes (*n*) are as follows: male sham (*n* = 64); female sham (*n* = 8); male pSNL (*n* = 79); female pSNL (*n* = 5). See also Figure S1. (D) FST immobility duration in male mice at baseline and WK4. A two-way ANOVA revealed a significant time × surgery interaction (F [1,35] = 18.82, ∗∗∗∗*p* < 0.0001). Fisher’s LSD post-hoc analysis confirmed that male pSNL mice exhibited significantly increased immobility at WK4 compared with both their baseline and sham controls (∗∗∗∗*p* < 0.0001). Data are presented as the mean ± SEM; violin plots indicate medians (solid lines) and quartiles (dashed lines). Male sham, *n* = 17; male pSNL, *n* = 20.

To establish a robust model of chronic pain and its affective consequences, mice were subjected to either pSNL or sham surgery, and behavioral outcomes were assessed over four weeks during their light phase, as outlined in the experimental timeline (Figures 1A and 1B). Mechanical paw sensitivity analysis revealed that pSNL surgery induced robust and long-lasting mechanical allodynia in male and female pSNL mice. As detailed in previous studies,^35^^,^^36^^,^^37^^,^^38^ we observed hypersensitivity related to acute post-operative pain resulting from the incision and muscle separation in sham-operated mice in week 1, as expected^35^^,^^36^^,^^37^^,^^38^ (∗∗∗∗*p* < 0.0001) (Figure 1C). By week 4, hypersensitivity resolved in the sham animals, while it persisted in the pSNL group (∗∗∗∗*p* < 0.0001), as also confirmed in previous work,^35^^,^^36^^,^^39^ indicating the transition to a chronic neuropathic state (Figures 1C and S1).

Notably, the development of passive coping in male mice was assessed using the forced swim test (FST). At week 4 post-surgery, pSNL mice exhibited a significant increase in immobility time compared with both their own baseline and sham-operated controls (∗∗∗∗*p* < 0.0001) (Figure 1D). This indicates the development of a passive coping behavior,^40^ a correlate of negative effect, which manifests during the chronic phase of the pain state, as reported previously.^41^^,^^42^^,^^43^ This temporal dissociation between immediate onset of pain and the delayed emergence of mood-related behavior is consistent with previous reports^41^^,^^42^^,^^43^ and provided a framework for investigating the underlying molecular changes in the VTA.

### Neuropathic pain associates with time-dependent VTA proteomic reprogramming

**Figure 2:**
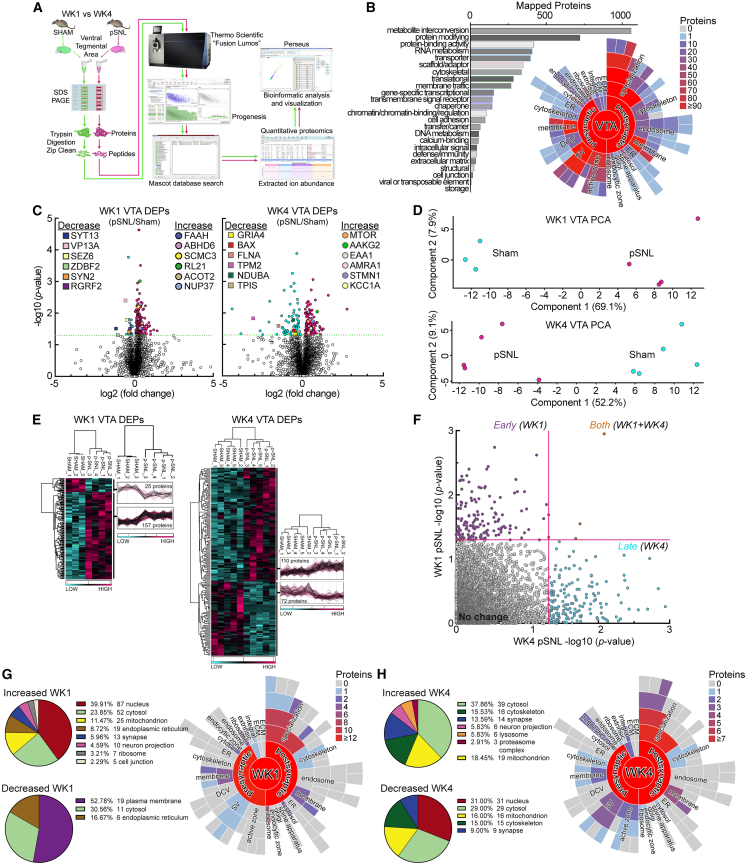
Neuropathic pain induces temporal remodeling of the VTA proteome (A) Workflow for the label-free quantitative proteomic analysis of VTA tissue at week 1 (WK1) and week 4 (WK4) post-surgery in male mice. Samples consisted of pooled bilateral tissue punches (2 punches/sample). Sample sizes: WK1 sham, *n* = 3 pooled samples; WK1 pSNL, *n* = 4 pooled samples; WK4 sham, *n* = 5 pooled samples; and WK4 pSNL, *n* = 5 pooled samples. (B) Functional classification of the 5,251 identified VTA proteins (PANTHER analysis and bar graph) and subcellular localization of 1,203 synaptic proteins (SynGO and starburst plot). (C) Volcano plots of differentially expressed proteins (DEPs) at WK1 and WK4. Insets highlight metabolic DEPs. WK1 includes lipid and endocannabinoid metabolism regulators (e.g., ACOT2 and ABHD6). WK4 includes energy and stress regulators from mTOR and AMPK pathways (e.g., MTOR and AAKG2) and mitochondrial complex I components (e.g., NDUAC and NDUBA). See also Table S2 and Figure S3. (D and E) Principal-component analysis (PCA) (D), and a heatmap from unbiased hierarchical clustering (E), demonstrating reproducible abundance shifts between male pSNL and sham groups at both time points. (F) Scatterplot comparing DEP significance at WK1 versus WK4, indicating two distinct molecular phases with minimal overlap. (G and H) Subcellular localization of DEPs at WK1 (G) and WK4 (H) mapped by Gene Ontology: Cellular Component (GO:CC) and SynGO. While proteins varied by time point, changes predominantly localized to overlapping compartments, including mitochondria, ribosomes, and the postsynaptic density. Annotation counts: WK1, 173 unique DEPs in GO:CC and 40 in SynGO; WK4, 127 unique DEPs in GO:CC and 30 in SynGO.

To define the molecular landscape of the VTA during the progression from acute to chronic pain in male mice, an unbiased, label-free quantitative proteomic screen was conducted on VTA tissue collected at week 1 and week 4 post-surgery (Figure 2A). Mass spectrometry identified 7,116 distinct proteins within the VTA proteome. PANTHER and Synaptic Gene Ontology (SynGO) analyses categorized these proteins by functional class and synaptic localization, respectively (Figure 2B). Subsequent bioinformatic analysis revealed a profound temporal remodeling of the VTA proteome (Figure 2C). Principal-component analysis (PCA) and hierarchical clustering revealed robust group separation at both time points, demonstrating that unambiguous biological signals persisted despite the necessity of tissue pooling required for anatomically constrained regions like the VTA (Figures 2D and 2E). This indicates two distinct molecular phases, a finding visualized in the scatterplot (Figure 2F) where the vast majority of significant proteins clustered along either the *x* axis (week 4) or *y* axis (week 1) but not on both axes. This demonstrated that the proteomic response at each time point involves largely separate, non-overlapping sets of proteins (Figure 2C; Table S2).

To determine where these proteomic changes occurred within the neuron, the subcellular localization of the differentially expressed proteins (DEPs) was analyzed using Gene Ontology (GO) and SynGO databases (Figures 2G and 2H). At both week 1 and week 4, the analysis revealed that the protein abundance changes like the post-synaptic density were concentrated in key cellular compartments, including mitochondria, ribosomes, and synaptic structures (Figures 2G and 2H). This observation indicates that while the specific proteins involved were distinct at each time point, the processes most consistently targeted shared similar functional locations within the neuron. Notably, among the proteins identified in the week 4 dataset were key components of the CaMKK2 signaling pathway, including CaMK1α and AMPK subunits (Figure 2C; Table S2), which are critical for responding to intracellular calcium signals and are implicated in synaptic remodeling.

**Figure 3:**
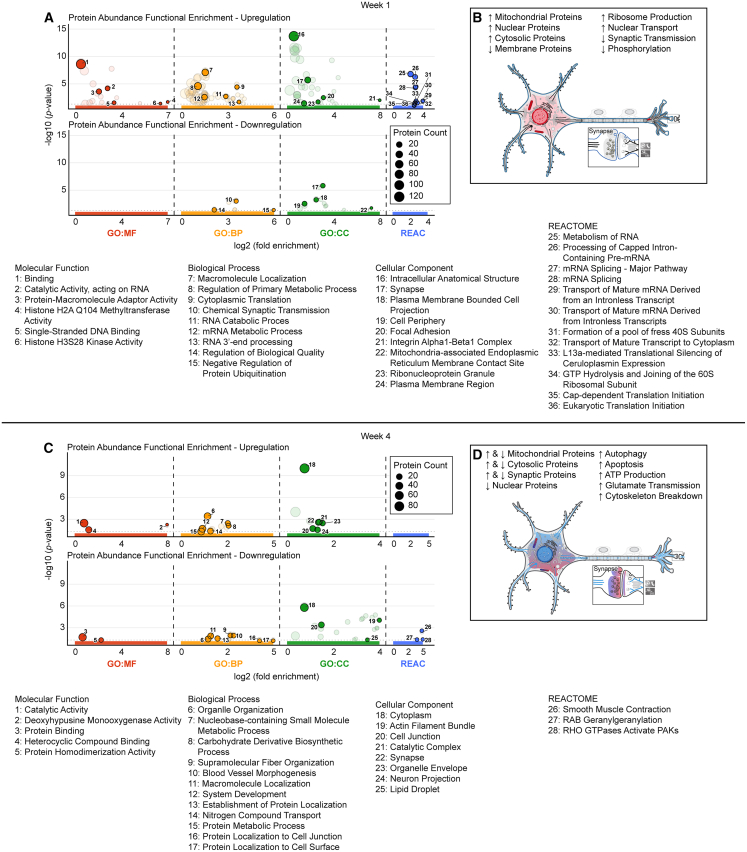
Summary of functional enrichment analysis reveals a two-phase molecular response in the VTA (A and C) Functional enrichment analysis (g:Profiler) summarizing differentially expressed proteins (DEPs) at week 1 (WK1) (A) and week 4 (WK4) (C), categorized by upregulated (top) and downregulated (bottom) proteins. Bubble plots display enriched GO (GO:MF, GO:BP, and GO:CC) and Reactome (REAC) terms, with driver terms highlighted to indicate the most significant or functionally relevant pathways and localizations. The *y* axis represents statistical significance (−log_10_ [*p* value]), the *x* axis represents fold enrichment, and bubble size corresponds to protein count. For a comprehensive list of all enriched terms (phosphorylation, RNA binding, Akt signaling, and signal transduction) as well as pathways (autophagy, glutamate transport, transporter trafficking, and mTOR signaling), see Figure S2. (B and D) Schematics of the major functional changes at WK1 (B) and WK4 (D). Red indicates enrichment related to increased protein abundance, while blue indicates enrichment related to decreased protein abundance across cellular spatial localizations. Neuron illustration adapted from SwissBioPics.^44^

At week 1, the proteomic profile indicated an early adaptive response specific to the male nerve injury mice. GO and Reactome pathway enrichment analyses revealed a significant upregulation of proteins involved in ribosome production, protein translation, and RNA binding (Figures 3A and 3B). Concurrently, there were changes in the abundance of proteins associated with mitochondrial metabolism and synaptic components (Figures 3A and S2; Table S2). Rather than a simple decrease in synaptic proteins, the data indicated changes in the abundance of proteins associated with synaptic vesicles and post-synaptic densities (Figures 3A and S2; Table S2), suggesting a reallocation of cellular resources.

By week 4, coinciding with the emergence of passive coping behavior, the VTA proteome shifted dramatically to a signature of severe cellular stress and maladaptation (Figures 3C and S2; Table S2). Enrichment analysis of the week 4 DEPs revealed a starkly different profile, with significant upregulation of pathways related to glutamate transmission, autophagy, and apoptosis, alongside a significant decrease in the abundance of proteins constituting the cytoskeleton (Figures 3C, 3D, and S2). These data delineate a two-phase molecular response in the VTA. The early phase appears to be an adaptive, high-energy attempt to cope with the persistent nociceptive signaling. However, the emergence of a distinct proteomic signature at week 4 indicates a shift to a potentially maladaptive state resembling metabolic failure and neuronal sensitivity.

### The chronic pain state is associated with a VTA phosphoproteome signature of cellular stress implicating CaMK1α signaling

**Figure 4:**
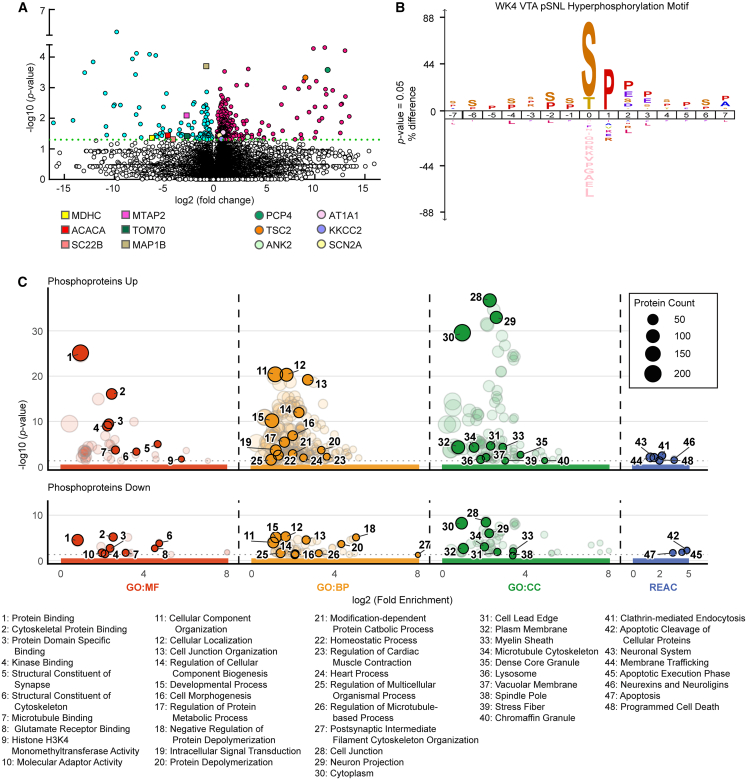
Widespread phosphorylation changes target postsynaptic architecture and neuronal viability pathways (A) Volcano plot of differentially phosphorylated peptides in the VTA at week 4 (WK4), demonstrating widespread hyperphosphorylation in male pSNL mice. (*n* = 3 pooled per group; 3 bilateral punches/sample). Data within the dashed rectangle were subsequently analyzed for iceLogo motif analysis. (B) iceLogo motif analysis of hyperphosphorylated sites (442 sites) revealing an enrichment for proline-directed phosphorylation. (C) Functional enrichment analysis (g:Profiler) of differentially phosphorylated proteins at WK4, separated by upregulated (“phosphoproteins up”) and downregulated (“phosphoproteins down”) proteins. Bubble plots display enriched GO (MF, BP, and CC) and Reactome (REAC) terms, with driver terms highlighted. The *y* axis represents statistical significance (−log_10_ [*p* value]), the *x* axis represents fold enrichment, and bubble size indicates protein count. For a detailed list of all enriched terms (including postsynaptic density, neuronal projection development, and calmodulin binding) and pathways (e.g., neuronal system programmed cell death), see Figure S2.

To further characterize the functional state of the VTA during chronic pain, a phosphoproteomic analysis was performed on VTA tissue from week 4 male pSNL and sham mice. The analysis revealed that pSNL induced widespread hyperphosphorylation, with 577 phosphopeptide ions showing significant alterations (Figures 4A and S2). To identify potential upstream kinases, an enrichment analysis of the phosphorylation motifs was performed. This revealed a significant enrichment for a proline-directed motif (Figure 4B), strongly implicating the activation of proline-directed kinase families, such as MAP kinases, which are multifunctional kinases involved in a diverse range of cellular functions and are classic mediators of cellular stress in chronic pain.^45^^,^^46^^,^^47^^,^^48^^,^^49^ These alterations included the differential phosphorylation of proteins integral to mitochondrial metabolism, glutamate transmission, autophagy, and neuronal structure, such as CaMKK2 and the cytoskeletal protein ANK2 (Figures 4C and S2; Table S2). Enrichment analysis of these differentially phosphorylated proteins strongly corroborated the themes identified in the proteome, showing alterations in proteins functionally associated with the neuronal projection assembly, post-synaptic densities, calmodulin binding, and glutamate signaling (Figures 4C and S2).

**Figure 5:**
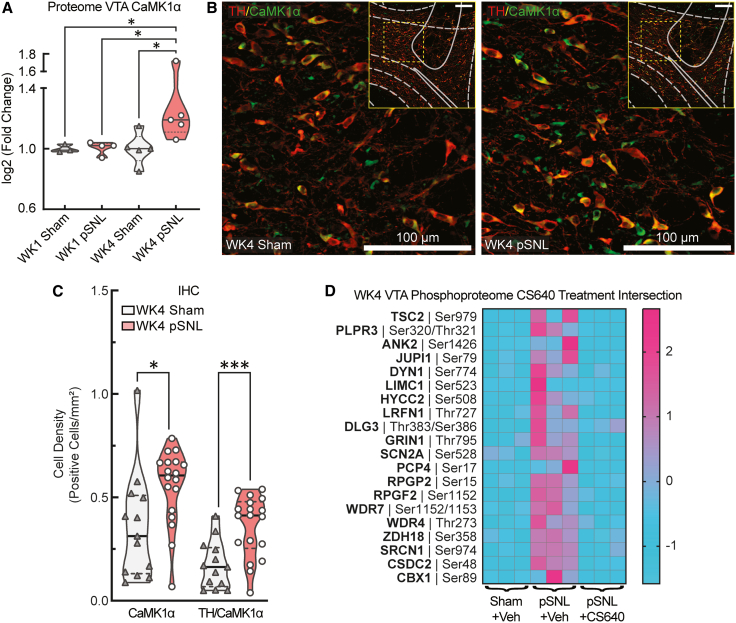
The chronic pain state is associated with CaMK1α pathway activation (A) Violin plot of CaMK1α protein abundance from the proteomic dataset, showing a significant increase in male WK4 pSNL mice (1-way ANOVA and Fisher’s LSD post-hoc: F [3,13] = 3.441, ∗*p* = 0.0488). Sample sizes: *n* = pooled biological replicates with 2 bilateral punches/sample; WK1 sham, *n* = 3; WK1 pSNL, *n* = 4; WK4 sham, *n* = 5; WK4 pSNL, *n* = 5. Violin plots indicate medians (solid lines) and quartiles (dashed lines). (B) Representative confocal images of tyrosine hydroxylase (TH, red) and CaMK1α (green) in the male VTA at WK4. Scale bars represent 100 μm. (C) Quantification of CaMK1α^+^ and TH^+^/CaMK1α^+^ cell densities, showing significant increases in pSNL mice (unpaired *t* tests: t(29) = 2.473, ∗*p* = 0.0195 and t(29) = 3.793, ∗∗∗*p* = 0.0007, respectively). Sham VTA slices, *n* = 13; pSNL VTA slices, *n* = 18; 2–3 VTA slices were analyzed per animal. Violin plots indicate medians (solid lines) and quartiles (dashed lines). (D) Acute CaMK1 inhibition reverses maladaptive phosphorylation shifts. Heatmap demonstrating that acute CaMK1 inhibition with CS640 (2 μg/μL, i.c.v.) reverses a subset of pSNL-induced phosphorylation changes in male mice at WK4. Sample sizes: *n* = pooled biological replicates; WK4 sham, *n* = 3; WK4 pSNL, *n* = 3; WK4 pSNL + CS640, *n* = 3; each sample contains 3 bilateral tissue punches.

Both the proteomic and phosphoproteomic datasets implicated the CaMKK2 signaling pathway, a critical regulator of cellular responses to the calcium influx. Specifically, the abundance of CaMK1α, a key downstream kinase in this pathway, was significantly increased in the VTA of week 4 pSNL mice (Figures 5A and S2). This finding was validated using IHC, which confirmed a significant increase in both the number of CaMK1α-positive cells (∗*p* = 0.0253) and the co-localization of CaMK1α within TH^+^ dopamine neurons (∗∗*p* = 0.0018) in pSNL mice (Figures 5B and 5C). To test the functional relevance of this pathway, the next-generation CaMK1 antagonist CS640^50^ was administered intracerebroventricularly. Acute inhibition of CaMK1 significantly reversed a subset of the pSNL-induced phosphorylation changes, particularly on proteins involved in neuronal structure and signaling (Figure 5D; Table S2). This focused analysis identified phosphosites that were both upregulated in pSNL injury and decreased after CS640 treatment, isolating pain state-specific CaMK1-dependent signaling. The upregulation of the CaMK1α pathway is consistent with our week 4 proteomic data, positioning CaMK1α as a key downstream effector of the signaling cascade in the VTA during chronic pain.

### Chronic pain induces convergent VTA pathologies: 2-AG depletion and Kv7 channel remodeling

**Figure 6:**
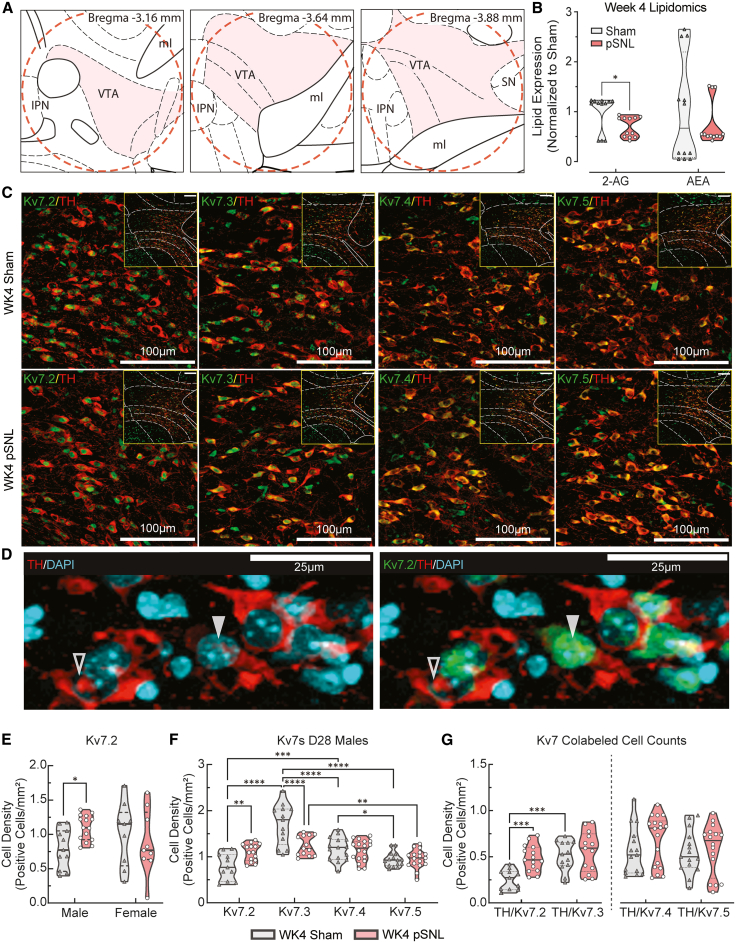
Convergent molecular pathologies in the VTA at week 4: 2-AG depletion and Kv7 channel remodeling (A) Schematic of midbrain coronal brain sections adapted from “*The Mouse Brain in Stereotaxic Coordinates*” (Franklin & Paxinos, 2007, 3rd edition),^59^ illustrating VTA regions evaluated by IHC (shaded) and approximate tissue punch size in proteomic and lipidomic studies (1 mm, dashed red circles). Mechanism 1: failure of endocannabinoid signaling. (B) VTA levels of 2-AG and AEA in male mice at week 4 (WK4). pSNL mice exhibited significantly lower 2-AG (Mann-Whitney U, ∗*p* = 0.0346), while AEA concentrations remained unchanged (Mann-Whitney U, *p* = 0.6181). Samples were run in triplicate. Sample sizes: *n* = pooled biological replicates; *n* = 4/group (pooled samples; 5 bilateral punches/sample: *n* = 4/group). Mechanism 2: remodeling of Kv7 channels. (C and D) Confocal photomicrographs of the VTA showing co-localization of TH (red) with Kv7.2–7.5 (green). Single-channel and overlay images illustrate subunit-specific expression patterns. In (D), co-expression (TH^+^/Kv7.2^+^) differentiates between cells expressing both markers versus those expressing only one (TH^+^/Kv7.2). Scale bars represent 100 μm (C) and 25 μm (D). (E) Sex-specific remodeling of Kv7.2 expression at WK4. Violin plots illustrate differences in Kv7.2 protein expression across sex and surgical groups in the VTA at WK4 post-surgery. A 2-way ANOVA revealed a significant sex × surgery interaction (F [1,40] = 4.134, ∗*p* = 0.0487). A Fisher’s LSD post-hoc demonstrated that Kv7.2 expression was significantly elevated exclusively in male pSNL mice compared to male sham controls (∗*p* = 0.0331), a remodeling effect that was absent in the female cohort. Sample sizes: *n* = VTA slices from 2–3 animals/group; sham male, *n* = 11, sham female, *n* = 9; pSNL male, *n* = 15, pSNL female, *n* = 9. (F) Kv7.2–7.5 subunit expression in male mice at WK4; 2-way ME ANOVA interaction (F [3,78] = 12.11, ∗∗∗∗*p* < 0.0001). Tukey’s post-hoc analysis revealed increased Kv7.2 (∗∗*p* = 0.0012) and decreased Kv7.3 (∗∗∗∗*p* < 0.0001) following pSNL. In male sham controls, Kv7.2 expression was significantly lower than those of Kv7.3 (∗∗∗∗*p* < 0.0001) and Kv7.4 (∗∗∗*p* = 0.0003). Sample sizes: *n* = VTA slices from 2–3 male mice/group; sham: Kv7.2, *n* = 11; Kv7.3, *n* = 13; Kv7.4, *n* = 15; Kv7.5, *n* = 15; pSNL: Kv7.2, *n* = 15; Kv7.3, *n* = 13; Kv7.4, *n* = 18; Kv7.5, *n* = 17. (G) Quantification of TH^+^/Kv7 co-labeled VTA cell densities. Quantification of co-localization between TH^+^ and Kv7.2–7.5 subunits. A 2W ME ANOVA revealed a significant surgery × subunit interaction (F [1,18] = 6.909, ∗*p* = 0.0170). Fisher’s LSD post-hoc analysis identified a significant increase in TH^+^/Kv7.2 co-localization (∗∗∗*p* = 0.0004), while TH^+^/Kv7.3 remained unchanged (*p* = 0.4830). Separate analyses of TH^+^/Kv7.4 and TH^+^/Kv7.5 showed no significant changes following pSNL (t(31) = 1.345, *p* = 0.1882 and t(30) = 0.6262, *p* = 0.5359, respectively). Sample sizes: *n* = VTA slices from 2–3 animals/group; sham: Kv7.2, *n* = 11; Kv7.3, *n* = 13; Kv7.4, *n* = 15; Kv7.5, *n* = 15; pSNL: Kv7.2, *n* = 15; Kv7.3, *n* = 13; Kv7.4, *n* = 18; Kv7.5, *n* = 17. Violin plots indicate medians (solid lines) and quartiles (dashed lines). See also Figures S4 and S5.

Prior clinical and preclinical studies associate chronic pain and mood disorders with dysregulation of both eCB and Kv7 channel activity.^5^^,^^8^^,^^9^^,^^12^^,^^13^^,^^51^^,^^52^^,^^53^^,^^54^ Therefore, we investigated the consequences of pSNL on these two systems in the VTA (Figure 6A). Proteomic analysis showed significant increases in eCB degradation enzymes in pSNL mice at week 1 (Figure S3; Table S2); however, these changes were no longer detectable at week 4 (ProteomeXchange: PXD072701). Despite this, liquid chromatography-mass spectrometry (LC-MS) analysis of pooled VTA punches at week 4 demonstrated a significant reduction in the eCB 2-AG in male pSNL mice relative to sham controls (∗*p* = 0.0162), while the levels of AEA remained unchanged (*p* = 0.4735) (Figure 6B). The stability of AEA levels, despite prior FAAH upregulation, is consistent with a compensatory increase in AEA synthesis that offsets enhanced degradation, although the underlying mechanisms remain to be defined.^55^^,^^56^ These VTA-specific findings mirror previously reported alterations in peripheral eCB levels associated with chronic pain and mood disorders.^16^^,^^17^^,^^57^^,^^58^

Concurrently, IHC analysis of Kv7 channel subunits, which mediate the inhibitory M current, was conducted in VTA sections from week 4 mice (Figures 6C–6G). This revealed a sex-specific remodeling of the Kv7.2 expression in male pSNL mice, which was not observed in female cohorts (Figures 6E and S4). Specifically, male mice at week 4 exhibited significant reorganization of the VTA M-current channel infrastructure, characterized by a significant increase in Kv7.2 expression (∗∗*p* = 0.0026) and a concomitant decrease in Kv7.3 expression (∗∗∗*p* = 0.0006) (Figure 6F).

To determine whether the observed Kv7.2 and Kv7.3 remodeling occurred specifically within dopamine neurons, we quantified subunit co-localization with TH^+^ cells (Figure 6G). We quantified VTA TH^+^ cell density in sections of male and female mice and found no significant differences across sex or surgery (Figure S5; *p* > 0.05), indicating that the TH^+^ dopamine neuron populations in the VTA are similar across groups and thus provide a stable TH^+^ baseline for co-labeling analyses. Because the neuronal M current is primarily generated by Kv7.2 and Kv7.3 heteromers,^22^^,^^23^^,^^24^^,^^27^^,^^60^^,^^61^^,^^62^ our analysis specifically tested the hypothesis of cell type-specific M-current remodeling. We, therefore, performed a focused 2-way ME ANOVA (surgery × subunit) restricted to the TH^+^/Kv7.2 and TH^+^/Kv7.3 co-localization data. While Kv7.4 and Kv7.5 were also quantified, they were analyzed separately as they are not principal components of the M current.

This stoichiometric shift was observed specifically within TH^+^ dopamine neurons (Figures 6G and S4). Notably, this molecular remodeling was absent in female mice, which exhibited no significant change in Kv7.2 expression following pSNL (Figure 6E). Together, these data reveal shifts in two convergent mechanisms that are likely to alter VTA excitability during chronic neuropathic pain. The depletion of 2-AG may disinhibit presynaptic terminals, increasing excitatory drive. Simultaneously, the dysregulation of Kv7 subunits in dopamine neurons—which favors the formation of functionally distinct Kv7.2 homomers— may impair a critical postsynaptic brake on neuronal firing (Figure 6G). This dual loss of inhibitory control provides a rational mechanistic basis for the proteins associated with cellular stress observed in our proteomic data.^12^^,^^13^^,^^63^

### Restoring 2-AG or Kv7 channel function reverses pain-induced passive coping behavior

**Figure 7:**
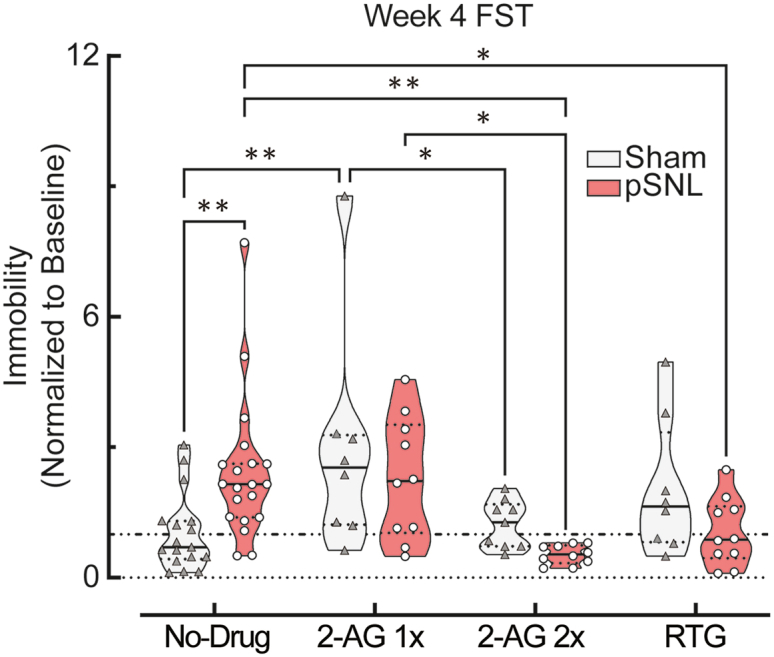
Pharmacological restoration of 2-AG or Kv7 channel function reverses passive coping behavior Results from the forced swim test (FST) in male mice at week 4 (WK4) post-surgery. The mice received systemic treatment of either two sequential doses of 2-AG (10 mg/kg, i.p.; administered 60 and 15 min prior to testing), a single dose of 2-AG (10 mg/kg, i.p.; administered 15 min prior to testing) or the Kv7 channel opener retigabine (RTG; 10 mg/kg, i.p.; 15 min prior). Data are normalized to baseline immobility. A 2W ME ANOVA followed by a Tukey’s multiple comparison test identified a significant interaction between treatment and pSNL-induced passive coping behavior (F [3,49] = 6.898, ∗∗∗*p* = 0.0006). Post-hoc analysis revealed that both the 2-AG sequential doses (∗∗*p* = 0.0011) and RTG (∗*p* = 0.0173) significantly reduced immobility time in male pSNL mice relative to their untreated WK4 FST. A single dose of 2-AG was found to be less effective (∗*p* = 0.0265). In the violin plot, solid lines depict the median, and dashed lines indicate quartiles. Sample sizes: *n* = total mice. Total cohort: male sham, *n* = 17; male pSNL, *n* = 20. Single dose 2-AG: sham, *n* = 8; pSNL, *n* = 10. Two sequential doses 2-AG: sham, *n* = 9; pSNL, *n* = 10. RTG: sham, *n* = 8; pSNL, *n* = 10.

To evaluate whether the observed dysregulation of 2-AG and Kv7 levels is linked to passive coping, we performed pharmacological rescue experiments. Male pSNL mice at week 4 were administered either systemic 2-AG or retigabine—a positive allosteric modulator of Kv7 channels—prior to the FST. Treatment with exogenous 2-AG significantly attenuated the increased immobility in the FST in pSNL mice in a dose-dependent manner. Similarly, administration of retigabine effectively reversed the passive coping behavior (∗∗∗∗*p* < 0.0001) (Figure 7). These results provide direct functional evidence that dysregulation of the 2-AG and Kv7 systems is causally involved in the expression of pain-induced passive coping behavior.

## Discussion

This study provides fundamental neurobiological insights into the mechanisms linking chronic pain with the development of passive coping behaviors.^40^^,^^64^ By generating a time-resolved molecular atlas of the VTA, we identified a shift from an initial adaptive response to a late-stage maladaptive state that aligns with the emergence of negative affect. The findings support a central model wherein chronic neuropathic pain alters the VTA molecular landscape. This transition may significantly impact neuronal excitability, metabolism, and structure and is strongly correlated with a convergent shift in 2-AG- and Kv7-mediated inhibitory control. Our data demonstrate that the resulting passive coping that can be rescued by pharmacologically targeting these specific pathways.

### A two-phase molecular response to neuropathic pain in the VTA

The temporal proteomic analysis delineates a distinct two-phase molecular response within the VTA. The week 1 profile, characterized by the upregulation of protein translation machinery and metabolic shifts, can be interpreted as an adaptive, albeit energetically costly, attempt to maintain neuronal integrity in the face of persistent nociceptive input. This increased capacity for protein synthesis likely reflects an attempt to support structural plasticity or mitigate early cellular stress.^65^^,^^66^ However, this high-energy state appears unsustainable. By week 4, the proteomic signature transitions toward pathways associated with autophagy, apoptosis, and cytoskeletal breakdown, signaling a shift toward metabolic failure. This molecular transition coincides with the emergence of passive coping behavior, suggesting that the exhaustion of initial adaptive mechanisms is a critical event in the development of pain-mood comorbidity.

### Convergent excitability mechanisms of VTA in chronic pain

A key mechanistic insight from this work is the identification of two convergent pathways that facilitate a state of VTA disinhibition. The profound deficit in VTA 2-AG at week 4 signifies the loss of a critical presynaptic brake. As a retrograde messenger, 2-AG acts on presynaptic CB1 receptors to suppress neurotransmitter release; its depletion, therefore, leads to disinhibition of inputs to the VTA neurons.^14^^,^^15^ Simultaneously, the VTA undergoes a stoichiometric remodeling of postsynaptic Kv7 channels. The observed shift from Kv7.3- to Kv7.2-enriched channels is functionally significant, as Kv7.2/7.3 heteromers generate a more robust M current at subthreshold potentials compared with Kv7.2 homomers.^22^^,^^24^^,^^28^^,^^31^^,^^61^^,^^62^ This shift would effectively weaken a postsynaptic brake that “normally” stabilizes membrane potential and limits repetitive firing.^29^^,^^32^^,^^67^^,^^68^

While these molecular findings strongly imply a shift in systems that regulate neuronal excitability, direct electrophysiological measurements are necessary to confirm the magnitude and direction of these functional changes in VTA neurons. Given the heterogeneous nature of VTA neurons, future studies need to use cell type-specific approaches to precisely map these excitability changes. Nevertheless, the convergent nature of our evidence—the downregulation of 2-AG and the shift in Kv7 subunit stoichiometry—supports that their simultaneous dysregulation is unlikely to be coincidental but instead reflects coordinated remodeling of VTA circuitry governing both pain-related and adaptive behavioral responses. Furthermore, pharmacological interventions targeting either pathway were sufficient to reverse the passive coping phenotype, providing compelling proof-of-concept for the functional relevance of these molecular targets.

### CaMK1α as a mediator of maladaptive plasticity

The upregulation and altered activation of CaMK1α represent a logical consequence of the proposed excitability changes. As a calcium/calmodulin-dependent kinase, its activation can serve as a molecular link between aberrant calcium influx and the downstream phosphorylation events that mediate cellular pathology.^69^^,^^70^ We demonstrated that acute CaMK1 inhibition with CS640 can reverse a subset of pain-induced phosphorylation changes, highlighting the functional relevance of this pathway. This inhibitor specifically reverses phosphorylation alterations in proteins essential to the core machinery of neuronal firing (SCN2A), cytoskeletal integrity (ANK2), and cellular stress signaling (TSC2). These findings position CaMK1α not only as a potential marker of altered neuronal structure but also as a key effector of maladaptive plasticity and a promising therapeutic target for mitigating cellular consequences of chronic pain.

In summary, this exploratory multi-omic study provides a comprehensive molecular survey of VTA adaptions in chronic neuropathic pain and its associated maladaptive coping behaviors. We identified temporally distinct molecular phases characterized by the remodeling of protein pathways related to excitability, metabolism, and neuronal structure. By validating the 2-AG and Kv7 systems as pivotal regulatory nodes, this work establishes a hypothesis-generating framework for treating the affective consequences of chronic pain. While many mechanistic details require further validation through targeted follow-up studies, this work represents a significant step toward understanding the molecular basis of pain-mood comorbidity. The identification of the 2-AG and Kv7 systems as effective therapeutic targets offers a promising path forward for developing treatments for the debilitating affective consequences of chronic pain.

### Limitations of the study

While this study provides a comprehensive preliminary multi-omic analysis, some limitations remain. First, tissue requirements for omic measurements necessitated sample pooling due to the relatively small size of the VTA. Although pooling can introduce systematic bias or mask individual variability, our PCA and hierarchical clustering demonstrated strong sample consistency and clear separation between experimental conditions, indicating a robust and reproducible proteomic dataset (Figures 2D and 2E).

Second, the use of whole VTA tissue punches lacks inherent cell type specificity. While we partially addressed this by using SynGO to analyze synapse-specific gene ontologies and IHC co-localization of selected proteins in dopamine neurons, our multi-omic analysis still reflects the heterogeneous VTA neuronal population. Consequently, cell type-specific effects cannot be assumed for all identified molecular changes, necessitating future single-cell or cell type-targeted analyses. Additionally, while the initial omics data are correlational, our pharmacological rescue experiments provide compelling evidence for a causal link between these molecular shifts and the observed behavior.

The discovery phase of our experimental design (proteomics, lipidomics, and FST) focused on male mice to establish a foundational model, a strategy predicated on substantial evidence that neuronal KCNQ/Kv7 channels exhibit profound sexual dimorphism.^30^^,^^71^^,^^72^^,^^73^ Preclinical models of KCNQ2 channelopathies, for instance, demonstrate male-specific behavioral deficits,^71^ while certain KCNQ3 mutations produce female-specific phenotypes.^72^ Furthermore, gonadal hormones are known to genomically remodel the M current by altering subunit expression, for example by suppressing KCNQ3 and upregulating KCNQ5,^30^^,^^73^ suggesting a compositionally distinct channel population in females.

By initially focusing on males, our approach avoided the confounding effects of pooling these distinct biological systems, which would likely have masked the precise adaptations identified here. Our subsequent validation in females confirmed the soundness of this strategy. The exclusive observation that Kv7 subunit remodeling occurs in male mice reinforces the biological necessity of the sex-stratified analysis (Figure 6E). Ultimately, these findings suggest that the molecular architecture of pain-mood comorbidity is fundamentally dimorphic, requiring distinct therapeutic strategies for each sex.

Finally, the use of a single behavioral assessment (i.e., FST) limits the scope of our evaluation regarding the global impact of chronic pain on mood. Future studies should include more diverse behavioral assessments to ensure treatment effects are thoroughly understood. Furthermore, while systemic pharmacological interventions successfully reversed passive coping, we did not directly measure whether 2-AG levels or Kv7 channel function were restored specifically within the VTA. Establishing the pharmacokinetic and metabolomic profiles of these systemically administered agents in the VTA remains a necessary step for future mechanistic studies.

## Resource availability

### Lead contact

Requests for further information, resources, and reagents should be directed to and will be fulfilled by the lead contact, Arthur C. Riegel (ariegel@arizona.edu).

### Materials availability

This study did not generate new unique reagents.

### Data and code availability

• Data: Mass spectrometry proteomics and phosphoproteomics data have been deposited to the ProteomeXchange Consortium via the PRIDE^74^ partner repository and are publicly available under the dataset identifier ProteomeXchange: PXD072701 and https://doi.org/10.6019/PXD072701. Original data have been deposited to Mendeley Data and are publicly available at Mendeley Data: https://doi.org/10.17632/smp9jdthxy.1.^75^
• Code: This paper does not report original code.
• Other items: Any additional information necessary for reanalysis is available from the lead contact upon request.

## Acknowledgments

This work was funded by grants from the 10.13039/100000026National Institute on Drug Abuse through the University of Arizona Center of Excellence in Addiction Studies (CEAS; P30DA051355), with additional support from the Department of Pharmacology Comprehensive Center for Pain and Addiction (CCPA) in the College of Medicine at the University of Arizona. The authors acknowledge the University of Arizona Cancer Center Analytical Chemistry Shared Resources (ACSR) for assistance with LC-MS services. The University of Arizona Health Sciences Quantitative Proteomics Laboratory is acknowledged for MS-based proteomic and phosphoproteomic support. This manuscript is the result of funding, in whole or in part, by the 10.13039/100000002National Institutes of Health (NIH). It is subject to the NIH Public Access Policy. Through acceptance of this federal funding, NIH has been given the right to make this manuscript publicly available in PubMed Central upon the official date of publication, as defined by NIH.

## Author contributions

C.C.D., L.A.M., P.R.L., T.W.V., and A.C.R. contributed to the design of the study; all authors participated in the acquisition of data; C.C.D., L.A.M., S.M.D.B., P.R.L., and A.C.R. contributed to the analysis of the data; C.C.D., P.R.L., and A.C.R. contributed to the interpretation of the data; C.C.D. and A.C.R. wrote the initial draft of the paper. All authors revised the paper for intellectual content and provided final approval of the paper to be published.

## Declaration of interests

The authors declare no competing interests.

## STAR★Methods

### Key resources table

**Table:** 

REAGENT or RESOURCE	SOURCE	IDENTIFIER
**Antibodies**		

Rabbit Polyclonal Anti-KCNQ2	Alomone (Jerusalem, ISR)	Cat.# APC-050; RRID:AB_2040101
Rabbit Polyclonal Anti-KCNQ3	Alomone (Jerusalem, ISR)	Cat.# APC-051; RRID:AB_2040103
Rabbit Polyclonal Anti-KCNQ4	Alomone (Jerusalem, ISR)	Cat.# APC-164; RRID:AB_2341042
Rabbit Polyclonal Anti-KCNQ5	Alomone (Jerusalem, ISR)	Cat.# APC-155; RRID:AB_2341038
Guinea Pig Polyclonal Anti-Tyrosine Hydroxylase	Synaptic Systems (Göttingen, DEU)	Cat.# 213-104; RRID:AB_2619897
Rabbit Monoclonal Anti-CaMKI	AbCam (Cambridge, GBR)	Cat.# ab68234; RRID:AB_1140889
Alexa Fluor 488 AffiniPure F(ab’)_2_ Fragment Donkey Anti-Rabbit IgG (H + L)	Jackson ImmunoResearch (West Grove, PA, USA)	Cat.# 711-546-152; RRID:AB_2340619
Cy3 AffiniPure Donkey Anti-Guinea Pig IgG (H + L)	Jackson ImmunoResearch (West Grove, PA, USA)	Cat.# 706-165-148; RRID:AB_2340460

**Chemicals, peptides, and recombinant proteins**		

Digitonin	Sigma Aldrich (Burlington, MA, USA)	Cat.#D141
Protease Inhibitor Cocktail	Sigma Aldrich (Burlington, MA, USA)	Cat.#P8340
100× Phosphatase inhibitor	Selleckchem (Houston, TX, USA)	Cat.#B15001
DAPI (4′,6-Diamidino-2-Phenylindole)	ThermoFisher (Waltham, MA, USA)	Cat.#D21490
Prolong Glass Antifade mountant	ThermoFisher (Waltham, MA, USA)	Cat.#P36980
d5-2-AG	Cayman Chemical (Ann Arbor, MI, USA)	Cat.# 362162
d4-AEA	Cayman Chemical (Ann Arbor, MI, USA)	Cat.# 10011178

**Critical commercial assays**		

Pierce BCA Protein Assay Kit	ThermoFisher (Waltham, MA, USA)	Cat.# 23225
High Select Phosphopeptide Enrichment Kits & Reagents	Thermo Scientific (Waltham, MA, USA)	Cat. #: A32992 & A32993
Pierce Quantitative Peptide Assays & Standards	Thermo Scientific (Waltham, MA, USA)	Cat. #: 23275

**Deposited data**		

Proteome Datasets	PRoteomics IDEntification Database (PRIDE)^74^	PXD072701 (https://www.ebi.ac.uk/pride/archive/projects/PXD072701) https://doi.org/10.6019/PXD072701
Original Data	Mendeley Data^75^	https://doi.org/10.17632/smp9jdthxy.1https://data.mendeley.com/datasets/smp9jdthxy/1

**Experimental models: Organisms/strains**		

Mouse: C57BL/6J	Jackson Labs (Waltham, MA, USA)	Strain #:000664 RRID: IMSR_JAX:000664

**Software and algorithms**		

Progenesis QI for proteomics (version 2.4)	Nonlinear Dynamics Ltd. (Newcastle Upon Tyne, UK)	https://www.nonlinear.com/progenesis/qi-for-proteomics/
Mascot (version 2.6)	Matrix Science (London, UK)	https://www.matrixscience.com/
Perseus	Tyanova et al.^76^ Tyanova & Cox^77^	https://maxquant.net/perseus/
DAVID	Sherman et al.^78^ Huang et al.^79^	https://davidbioinformatics.nih.gov/
SynGO	Koopmans et al.^80^	https://syngoportal.org/
iceLogo	Colaert et al.^81^	http://plogo.uconn.edu/
Xcalibur (version 2.1.0)	Thermo Scientific (Waltham, MA, USA)	Cat. #: OPTON-30967
Scaffold (version Scaffold_4.8.7)	Proteome Software (Portland, OR, USA)	https://www.proteomesoftware.com/
GraphPad Prism (version 10.4.2)	GraphPad Software (San Diego, California, USA)	https://www.graphpad.com/
Olympus FluoView FV10-ASW	Olympus (Tokyo, JPN)	N/A
FIJI/ImageJ (version 1.54p)	Schindelin et al.^82^	https://imagej.net/software/fiji/
g:Profiler	Kolberg et al.^83^ Kolberg et al.^84^	https://biit.cs.ut.ee/gprofiler/gost
R	R Core Team, 2025^85^	https://www.r-project.org/

**Other**		

Orbitrap Fusion Lumos Tribrid Mass Spectrometer	Thermo Scientific (Waltham, MA, USA)	Cat.# IQLAAEGAAPFADBMBHQ
EASY-Spray Source	Thermo Scientific (Waltham, MA, USA)	Cat. #: ES081
UltiMate 3000 RSLCnano System	Thermo Scientific (Waltham, MA, USA)	Cat. #: ULTIM3000RSLCNANO
EASY Spray C18 LC Column	Thermo Scientific (Waltham, MA, USA)	Cat. #: ES906
Solid Phase C18 ZipTip	MilliporeSigma (Billerica, MA, USA)	Cat. #: ZTC18S
SM2010R Microtome	Leica (Wetzlar, DEU)	N/A
Olympus FluoView FV1200 System	Olympus (Tokyo, JPN)	N/A
6495C Triple Quadrupole Mass Spectrometer	Agilent (Palo Alto, CA, USA)	N/A
1290 Infinity II UPLC System	Agilent (Palo Alto, CA, USA)	N/A
Acquity UPLC BEH C-18 1.7 μm 2.1 × 100 mm column	Waters (Milford, MA, USA)	SKU: 186002353

### Experimental model and study participant details

#### Animals

A total of 154 mice (138 males and 16 females, C57BL/6J; Jackson Labs, MA), aged 7–8 weeks, were utilized in this study. Mice were housed in standard cages with 3–5 animals per cage in a climate-controlled room under a 12-hour light-dark cycle, with *ad libitum* access to food and water. All experimental procedures were conducted during the light phase and were approved by the University of Arizona Animal Care Use Committee (Protocols 19-556, 19-600, and 2021-0804), in strict compliance with NIH guidelines for laboratory animal care.

### Method details

#### Partial sciatic nerve ligation (pSNL) surgery

Neuropathic pain was induced via pSNL (n=80 pSNL; n=74 Sham). Mice were anesthetized with isoflurane, and the sciatic nerve was exposed via a 2 mm incision at the lateral aspect of the thigh. Approximately 1/3 to 1/2 of the nerve diameter was tightly ligated using 9-0 nylon suture. In sham-operated controls, the nerve was exposed but not manipulated. Muscle and skin were closed using absorbable 5-0 polyglycolic and non-absorbable 6-0 polypropylene sutures, respectively.

##### Behavioral assessments

Mechanical Allodynia: Sensitivity was evaluated using the von Frey (vF) test at baseline, Week 1 (WK1), and Week 4 (WK4) post-surgery to monitor the development and persistence of allodynia. Passive Coping: The Forced Swim Test (FST) was employed at WK4 to assess maladaptive coping behaviors. Immobility duration was recorded and normalized to baseline values to evaluate chronic pain-induced shifts and subsequent pharmacological reversal.

Pharmacological Interventions: Systemic Modulation: Retigabine (10 mg/kg, IP) was administered 15 minutes prior to testing in a 0.9% saline vehicle. 2-AG (10 mg/kg, IP) was administered in a 1:1:8 DMSO:Tween80:saline vehicle. A “sequential dose” regimen (administered 60 and 15 min prior to testing) was utilized to address the short biological half-life of 2-AG. CaMK1 Inhibition: Acute ICV infusion of CS640 (2ug/ul) was performed at WK4 to evaluate the reversal of pain-induced phosphorylation alterations.

#### Drugs

The dose of retigabine (10 mg/kg, IP) was selected based on previous studies demonstrating effective modulation of Kv7 channel activity and behavioral outcomes in rodent models.^9^^,^^86^

While 2-AG has a short half-life,^87^ systemic administration of exogenous 2-AG has shown robust behavioral effects in preclinical rodent models.^88^^,^^89^ We used doses (10 mg/kg, IP) and vehicle formulations (1:1:8 DMSO:Tween80:saline for 2-AG; 0.9% saline for retigabine) consistent with published protocols demonstrating central behavioral effects.

#### Behavioral tests

##### Mechanical withdrawal (tactile allodynia)

Tactile allodynia was assessed at baseline and at Week 1 and Week 4 post-surgery by measuring the paw withdrawal threshold in response to probing with a series of calibrated von Frey filaments, which serve as innocuous stimuli. Prior to testing, mice were acclimated in suspended wire-mesh cages for 60 minutes. Filaments were applied perpendicularly to the plantar surface of the hindpaw for 2 seconds. A positive response was characterized by a sharp withdrawal of the paw during the initial application of the filaments, which ranged in intensity (2.44, 2.83, 3.22, 3.61, 4.08, 4.31, and 4.56 g).

Paw withdrawal thresholds were determined using the non-parametric “up-and-down” method^90^^,^^91^ described by Dixon (1980),^92^ in which the stimulus intensity was incrementally increased until a positive response was observed, after which it was decreased until a negative response occurred. This protocol was repeated until three behavioral changes were noted. The 50% paw withdrawal threshold (50% PWT) was calculated using the following formula: 10[Xf+kδ])/10,000, where Xf where Xf represents the value of the last von Frey filament employed, k is the Dixon value corresponding to the positive/negative response pattern, and δ is the logarithmic difference between stimuli. To ensure rapid and accurate calculation of 50% PWT, data were processed via a dedicated web application (https://bioapps.shinyapps.io/von_frey_app/). Tactile allodynia was defined as a significant reduction in the withdrawal threshold compared to the pre-treatment baseline.

##### Passive coping (forced swim test)

Coping behavior was assessed between 28 and 35 days post-surgery in male mice by measuring immobility time in a water-filled cylindrical container (maintained at 25°C) for a duration of 5 minutes. To minimize the total number of exposures and mitigate potential carryover effects associated with repeated testing (e.g., increased baseline immobility),^93^ assessments were restricted to two time points: a pre-surgery baseline and a 4-week post-surgery evaluation. The 4-week interval was selected based on established literature^41^^,^^42^^,^^43^ indicating that maladaptive passive coping behaviors associated with chronic pain typically emerge by this stage.^41^^,^^42^^,^^43^ Total immobility time across the 5-minute test duration served as the quantitative measure of passive coping behavior.

##### Pharmacological rescue and dosing logic

To evaluate the functional reversal of passive coping, two distinct treatment cohorts were utilized: Standard Regimen: One cohort received 2-AG (10 mg/kg, IP; prepared in 1:1:8 DMSO:Tween 80:saline) 32 days post-surgery, or retigabine (RTG; 10 mg/kg, IP; prepared in 0.9% saline) 35 days post-surgery These compounds were administered 15 minutes prior to the FST. Bioavailability Optimization (Double Dose): A second cohort received two doses of 2-AG (10 mg/kg, IP) 31 days post-surgery, administered at 60 and 15 minutes prior to the FST. This “double dosing” strategy was specifically employed to ensure adequate substrate bioavailability and target engagement throughout the testing window, accounting for the rapid metabolic degradation of exogenous 2-AG.

#### Proteomic analyses

##### Tissue collection and homogenization

Ventral tegmental area (VTA) tissue was collected using previously published procedures,^86^^,^^94^^,^^95^ pooled VTA samples from 1 and 4 weeks post-surgery. Using a chilled brain matrix, 1 mm thick coronal sections were prepared to identify the VTA (AP: -3.16 to -3.88 mm) as the region medial to the compact part of the substantia nigra while excluding the medial lemniscus. Samples were collected on ice with a 1 mm biopsy punch, snap-frozen on dry ice, and stored at -80°C. To ensure sufficient protein for mass spectrometry, bilateral punches from separate animals were pooled (2 to 5 bilateral punches per sample).

Pooled samples were incubated on ice for 15 minutes in Radioimmunoprecipitation assay (RIPA) buffer (20 mM Tris HCL, 150 mM NaCl, 2 mM EDTA, 0.1% SDS, 1% TritonX-100, 0.25% Deoxycholate, 1 mM Na Orthovanadate, 1 mM phenylmethylsulfonyl fluoride) supplemented with 1X protease inhibitor, 1X phosphatase inhibitor, and 1% Digitonin . Samples were homogenized via ultrasonication and centrifuged at 15,000g for 10 minutes at 4°C. Protein concentration in the resulting supernatant was determined using a Pierce BCA protein assay kit.

##### In-gel digestion and peptide purification

For proteome-wide experiments, 50 micrograms of mouse VTA lysate was separated by SDS-PAGE, and each lane was partitioned into seven slices. Gel slices underwent trypsin digestion and subsequent peptide purification via C18-based desalting as previously described.^94^^,^^95^ Briefly, gel slices were placed in 0.6 mL LoBind tubes, destained twice with 375 microliters of 50% acetonitrile (ACN) in 40 mM NH4HCO3, and dehydrated with 100% ACN for 15 minutes. Following aspiration, gel pieces were dried in a vacuum centrifuge at 60°C for 30 minutes. Trypsin (250 ng) in 20 microliters of 40 mM NH4HCO3 was added and maintained at 4°C for 15 minutes before adding 50 to 100 microliters of 40 mM NH4HCO3. Digestion proceeded overnight at 37°C and was terminated with 10 milliliters of 5% formic acid (FA). After a 30-minute incubation at 37°C and 1-minute centrifugation, the supernatant was transferred to a clean LoBind tube. A second extraction was performed using 40 milliliters of 0.5% FA; the extracts were combined and dried to approximately 5 to 10 milliliters.

Samples were incubated at room temperature for 15 minutes following the addition of 10 microliters of 0.05% heptafluorobutyric acid/5% FA (vol/vol). Peptide mixtures were loaded onto a solid-phase C18 ZipTip , washed with 35 milliliters of 0.005% heptafluorobutyric acid/5% FA (vol/vol), and eluted sequentially with 4 milliliters of 50% ACN/1% FA (vol/vol) and 4 milliliters of 80% ACN/1% FA (vol/vol). Combined eluates were dried completely by vacuum centrifugation and resuspended in 6 milliliters of 0.1% FA (vol/vol) followed by 2 minutes of sonication. Finally, 2.5 milliliters of the sample was analyzed by mass spectrometry.

##### Mass spectrometry

HPLC-ESI-MS/MS was performed in positive ion mode on a Thermo Scientific Orbitrap Fusion Lumos tribrid mass spectrometer fitted with an EASY-Spray Source (Thermo Scientific) as previously described.^95^ In brief, NanoLC was performed using a Thermo Scientific UltiMate 3000 RSLCnano System with an EASY Spray C18 LC column (Thermo Scientific, 50 cm x 75 mm inner diameter, packed with PepMap RSLC C18 material, 2 mm, cat. # ES803); loading phase for 15 minutes; mobile phase, linear gradient of 1–47% ACN in 0.1% FA for 106 minutes, followed by a step to 95% ACN in 0.1% FA over 5 minutes, hold 10 minutes, and then a step to 1% ACN in 0.1% FA over 1 minute and a final hold for 19 minutes (total run 156 minutes); Buffer A = 100% H_2_O in 0.1% FA; Buffer B = 80% ACN in 0.1% FA; flow rate, 300 nL/min. All solvents were liquid chromatography mass spectrometry grade. Spectra were acquired using Xcalibur, version 2.1.0 (Thermo Scientific). A “TopSpeed” data-dependent MS/MS analysis was performed (acquisition of a full scan spectrum followed by collision-induced dissociation mass spectra of the Top N most intense precursor ions within the 3 second cycle time). Dynamic exclusion was enabled with a repeat count of 1, a repeat duration of 30 seconds, an exclusion list size of 500, and an exclusion duration of 40 seconds.

##### Label-free Quantitative Proteomics

Progenesis QI for proteomics software (version 2.4, Nonlinear Dynamics Ltd., Newcastle upon Tyne, UK) was used to perform ion-intensity based label-free quantification similar to as previously described.^95^ In brief, in an automated format, .raw files were imported and converted into two-dimensional maps (y-axis = time, x-axis =m/z) followed by selection of a reference run for alignment purposes. An aggregate data set containing all peak information from all samples was created from the aligned runs, which was then further narrowed down by selecting only +2, +3, and +4 charged ions for further analysis. A peak list of fragment ion spectra was exported in Mascot generic file (.mgf) format and searched against the *Mus musculus* SwissProt database (17079 entries) using Mascot (Matrix Science, London, UK; version 2.6). The search variables that were used were: 10 ppm mass tolerance for precursor ion masses and 0.5 Da for product ion masses; digestion with trypsin; a maximum of two missed tryptic cleavages; variable modifications of oxidation of methionine and phosphorylation of serine, threonine, and tyrosine; ^13^C=1. The resulting Mascot .xml file was then imported into Progenesis, allowing for peptide/protein assignment, while peptides with a Mascot Ion Score of <25 were not considered for further analysis. Precursor ion-abundance values for peptide ions were normalized to all proteins. Significant protein abundance values were identified using one-way ANOVA with a p-value threshold of < 0.05. Samples were filtered to include only those with at least 2 unique peptide sequences, and Z-score normalization was subsequently applied in Perseus. Unbiased hierarchical clustering analysis (heat map) and principal component analysis (PCA) were conducted using Perseus.^76^^,^^77^ Heat map and PCA analysis serve to visualize the similarity of individual samples by their proteomic profiles. PCA specifically shows clusters of individual samples indicating these similar proteomic profiles mapped by their greatest variance in the dataset (PC1) and their second greatest variance in the dataset (PC2). Gene ontology (GO) and Reactome pathway enrichment analyses were completed with g:Profiler^83^^,^^84^ and the DAVID tool.^78^^,^^79^ Driver terms were identified using g:Profiler's enrichment analysis with a significance threshold of p < 0.05 (adjusted for multiple testing using the g:SCS algorithm). For synapse-specific subcellular localization analysis, SynGO was utilized.^80^ Phosphorylation sequence enrichment analysis was performed using the iceLogo generation tool.^81^ Volcano plots and scatter plots were created in GraphPad Prism.

#### Phosphoproteomics

##### Intracerebroventricular (ICV) administration

To evaluate differences in protein phosphorylation abundance following pSNL surgery and CS640 treatment, mice were anesthetized and received a 5 microliter ICV injection of CS640 (2 micrograms/microliter) or vehicle (1:1:8 DMSO:Tween 80:saline solution). Injections were administered at a rate of 1 microliter/sec using a Hamilton syringe. ICV administration was selected over direct VTA infusion to minimize mechanical tissue damage that could confound phosphoproteomic results. Experimenters were blinded to surgical and treatment group assignments during drug administration.

##### Tissue processing and phosphopeptide enrichment

Bilateral VTA tissue was collected 30 minutes post-injection. For each sample, 200 to 400 micrograms of VTA protein lysate underwent in-solution tryptic digestion. Phosphopeptides were enriched using metal oxide affinity chromatograph according to the manufacturer’s protocol (Thermo Scientific), utilizing methods similar to those previously described.^96^^,^^97^

##### Sample preparation for mass spectrometry

The resulting dried peptides were resuspended in 20 microliters of 0.1% formic acid (v/v). Peptide concentrations were determined using a Pierce Quantitative Colorimetric Peptide Assay Kit (Thermo Scientific) per the manufacturer’s instructions. Finally, 350 nanograms of the purified sample was analyzed by mass spectrometry.

#### Immunohistochemistry

##### Tissue preparation and sectioning

Mice were deeply anesthetized with isoflurane and underwent transcardial perfusion with cold 0.1 M phosphate-buffered saline (PBS), followed by cold 4% paraformaldehyde (PFA) in PBS. Brains were extracted, post-fixed in 4% PFA for 24 hours, and then cryoprotected in 30% sucrose in 0.1 M PBS for 48 hours. Midbrain coronal sections (25 micrometers thick) were obtained using a SM2010R Leica microtome. The VTA was identified in free-floating sections as the region medial to the substantia nigra pars compacta, excluding the medial lemniscus (AP: -3.16 to -3.88 mm).

##### Antigen retrieval and blocking

Selected tissue slices were washed in 4% PFA for 20 minutes, followed by three rinses in 0.1 M PBS. Antigen retrieval was performed at 90°C for 60 minutes in 10 mM sodium citrate buffer (0.05% Tween-20, pH 6.0). Following retrieval, sections were washed twice with PBS containing Triton X-100 (PBS-TX100) and once with PBS. Tissue was then incubated for 1 hour in a blocking solution containing 3% bovine serum albumin (BSA), 0.3% Triton X-100, 0.3% Tween-20, 0.1% sodium azide, and 3% normal donkey serum in 0.1 M PBS.

##### Antibody incubation and mounting

VTA sections were incubated in primary antibody solutions overnight at 4°C with gentle oscillation. Primary antibodies included: Anti-KCNQ2 (1:800, Rabbit, Alomone), Anti-KCNQ3 (1:800, Rabbit, Alomone), Anti-KCNQ4 (1:400, Rabbit, Alomone), Anti-KCNQ5 (1:200, Rabbit, Alomone) Anti-CaMKIa (1:500, AbCam), Anti-Tyrosine Hydroxylase (TH) (1:800, Guinea Pig, Synaptic Systems).

Following primary incubation, samples were washed in PBS-TX100 and incubated with secondary antibodies for one hour at room temperature. Secondary antibodies used were anti-Guinea Pig Cy3 (1:600, Jackson ImmunoResearch) and anti-Rabbit AF488 (1:400, Jackson ImmunoResearch). Sections were counterstained with DAPI, mounted onto glass slides using Prolong Glass Antifade mountant, and allowed to harden overnight prior to confocal imaging.

#### Imaging and analysis

##### Confocal photomicrograph acquisition

Photomicrographs were acquired using an Olympus Fluoview FV1200 confocal microscope equipped with Olympus FV10-ASW software (v4.02). Images were captured at 20x magnification (NA 0.8) with a 1024 x 1024 pixel resolution. To ensure uniformity and allow for valid quantitative comparisons, all software and hardware settings—including laser power, gain, and offset—were held constant for each specific antibody within each staining session.

##### Region of interest (ROI) delineation

The anatomical boundaries of the ventral tegmental area (VTA) were delineated by overlaying coronal section schematics from the mouse brain atlas (Franklin & Paxinos, 3rd Edition, 2007)^59^ onto the captured images. For each specimen, analysis was performed on 2-3 VTA slices located contralateral to the surgical site. Slices were anatomically matched across all animals to minimize variance along the anterior-to-posterior axis of the VTA.

##### Automated image processing and quantification

Image analysis was conducted using the FIJI/ImageJ software^82^ platform. A custom FIJI macro was developed to standardize the quantification of protein-positive cells within the defined VTA ROI. This macro utilized a consistent sequence of image processing steps to ensure accuracy and reproducibility, including: Linear adjustments: Application of a Gaussian blur to reduce high-frequency noise; Nonlinear adjustments: Gamma correction, auto-thresholding, rolling-ball background subtraction, and maximum intensity Z-projection.

These standardized processing steps were applied to whole images prior to cell counting. Quantitative values were derived for TH+, Kv7+, TH+/Kv7+, CaMKIa+, and TH+/CaMKIa+ populations. Final cell densities represent the total number of positive cells identified within the area of the standardized VTA ROI per section.

#### Lipidomic analysis

##### Quantification of 2-AG and AEA by LC-MS

Following methodologies established in prior research,^98^ tissue samples (n = 5 bilateral punches per sample) were subjected to organic solvent extraction to purify for LC-MS analysis, following the protocol detailed by Wilkerson et al.^20^ On the day of processing, tissues were weighed and homogenized using a Dounce homogenizer with 1 ml of chloroform/methanol (2:1 v/v), supplemented with phenylmethylsulfonyl fluoride (PMSF, 1 mM) to inhibit degradation by endogenous enzymes. The homogenates were subsequently mixed with 0.3 ml of NaCl (0.7% w/v), vortexed, and centrifuged at 3,200 × g for 10 minutes at 4°C. The aqueous phase, along with debris, was collected and subjected to two additional extractions with 0.8 ml of chloroform. The organic phases were combined, and an internal standard was introduced to each sample. Mixed internal standards were prepared through serial dilution of d4-AEA (Cayman Chemical, MI) and d5-2-AG (Cayman Chemical, MI) in acetonitrile to facilitate concentration calculations and to account for run variability according to Wilkerson et al.^20^ The organic solvents were evaporated using nitrogen gas, following which glycerol in methanol (6 μl, 30%) was added prior to evaporation. The dried samples were reconstituted with 0.2 ml of chloroform and combined with 1 ml of ice-cold acetone. The mixtures were then centrifuged at 1,800 × g for 5 minutes at 4°C, after which the organic layer of each sample was collected and subjected to further nitrogen evaporation.

Analysis of 2-AG and AEA was conducted using an Agilent 6495C triple quadrupole mass spectrometer coupled with a 1,290 Infinity II UPLC system (Agilent, Palo Alto, CA). The system was used in the electrospray positive mode with a gas temperature of 150°C, a flow rate of 5 L/min, a nebulizer pressure of 15 psi, a capillary voltage of 4,500 V, sheath gas set to 400°C at a flow rate of 12 L/min, and a nozzle voltage of 300 V. Monitored transitions included 348.3 → 287.3 and 62, 352.3 → 287.4 and 65.9, 379.3 → 287.2 and 269.2, and 384.3 → 287.2 and 296.1 for AEA, 2-AG, d4-AEA, and d5-2-AG, respectively. The first fragment was utilized for quantification, while the second served for confirmation. The initial 3 minutes of analysis were diverted to waste. Chromatographic separation was accomplished using an isocratic system comprising 21% 1 mM ammonium fluoride and 79% methanol on an Acquity UPLC BEH C-18 1.7 μm 2.1 × 100 mm column (Waters, Milford, MA) maintained at 60°C. Following each injection, the column was washed with 90% methanol for one minute and re-equilibrated for 5 minutes prior to the next injection. Samples were stored at 4°C. Calibration solutions were prepared from serial dilutions of AEA and 2-AG stock solutions in 80% C_2_H_3_N. Calibration curves for each analysis were generated by adding 10 μl of internal standard solution to 20 μl of standard solution. To the dried samples, 200 μl of a solvent mixture containing 80:20 C_2_H_3_N:H_2_O was added, followed by vortexing and sonication. Samples underwent centrifugation at 15,800 × g for 5 minutes at 4°C, whereupon the supernatant was transferred to autosampler vials, and 5 μl was injected for analysis.

### Quantification and statistical analysis

Statistical analyses were performed using GraphPad Prism 10.6.1 (La Jolla, CA). Data are presented as mean ± SEM, and statistical significance was pre-defined at p < 0.05. Analysis of variance (ANOVA) was employed for multi-group comparisons, including one-, two-, and three-way designs, utilizing mixed-effects models where missing values or specific repeated measures occurred. Post-hoc analyses were selected based on the specific experimental design and the nature of the biological questions being addressed: (i) Tukey's Multiple Comparison Test was utilized for datasets requiring all-to-all pairwise comparisons across multiple time points or treatment conditions (e.g., Figures 1C, 6F, and 7) to control the family-wise error rate; (ii) Fisher's LSD (Least Significant Difference) was employed for planned comparisons following a significant main effect or interaction, when testing pre-defined biological hypotheses including the sex-specific differences and individual subunit remodeling (e.g., Figures 1D, 5A, and 6E, and the M-current component—Kv7.2 and Kv7.3—in Figure 6G); (iii) Student's t-tests were used for simple two-group comparisons (Figure 5C and non-M-current component—Kv7.4 and Kv7.5—in Figure 6G) , while non-parametric Mann-Whitney U tests were applied to lipidomic data (Figure 6B) where normal distribution could not be assumed. Similar statistical approaches were applied to supplemental datasets where applicable. Functional enrichment analysis and additional data visualizations were performed using R (version 4.5.2),^85^ the gprofiler2 package version 0.2.3,^84^ and the ggplot2 package version 4.0.0.^99^ For proteomic and phosphoproteomic analyses, statistical significance of differentially expressed proteins (DEPs) and phosphopeptides was determined as described in the Label-free Quantitative Proteomics methods.

## Supplemental information

Document S1. Figures S1–S5

Table S1. Summary of statistical analyses, related to Figures 1, 5, 6, and 7

Table S2. Differentially expressed proteins identified in proteomic and phosphoproteomic analyses, related to Figures 2, 3, 4, and 5
