# Supplementary material for: Temporal multi-omic exploration of the ventral tegmental area in chronic pain and passive coping behaviors
Source: iScience. 2026 May 22;29(6):116085. doi: 10.1016/j.isci.2026.116085 (PMC13224009; doi:10.1016/j.isci.2026.116085)
Supplement: Document S1. Figures S1–S5 [file mmc1.pdf]

iScience, Volume *29*

Supplemental information

Temporal multi-omic exploration

# of the ventral tegmental area

in chronic pain and passive coping behaviors

Cody C. Diezel, Lisa A. Majuta, Erfan Bahramnejad, Kelly L. Karlage, Jennifer L. Partin, Saniya M.D. Barbour, Ingrid L. Peterson, Matthew Flowers, Sophia T. von Hippel, Riley Haveman, Ethan Villarroel, Isabella Villarroel, Paul R. Langlais, Tally M. Largent-Milnes, Todd W. Vanderah, and Arthur C. Riegel

Supplemental Figures

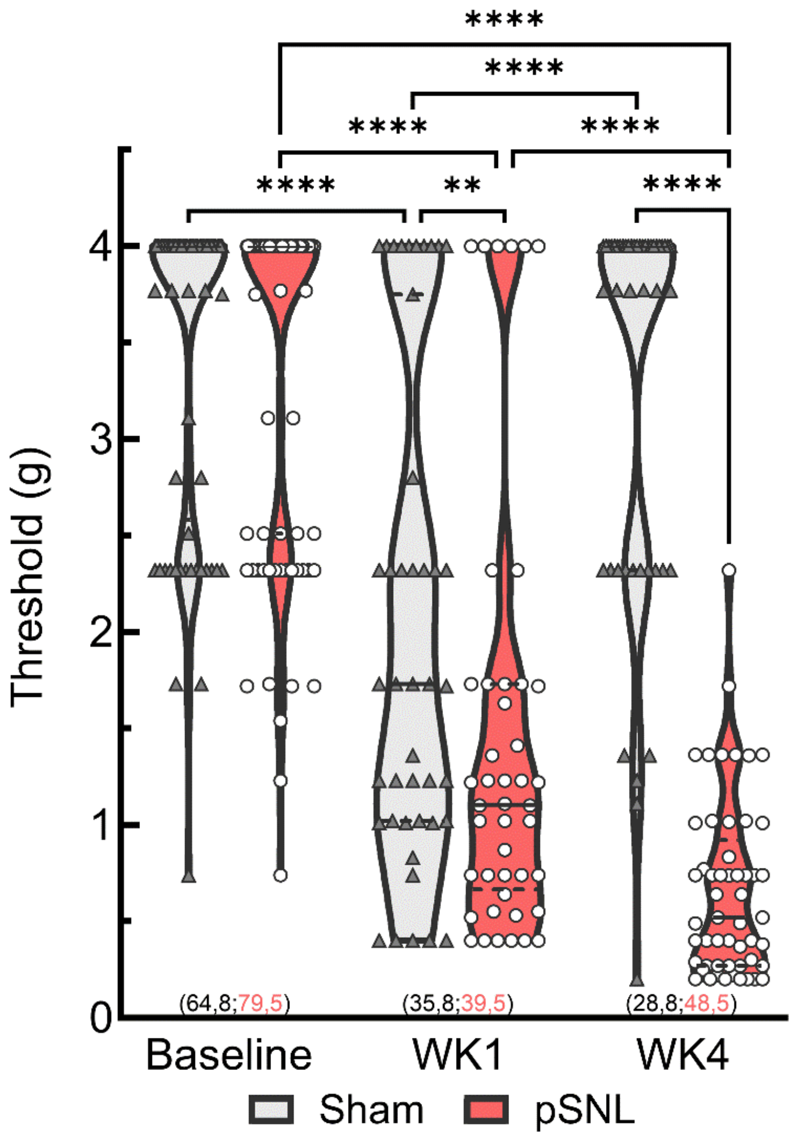

Supplementary Figure S1. pSNL Surgery Causes Long-Lasting Allodynia. Violin plot of paw withdrawal thresholds in male and female mice. A significant time x surgery interaction was observed (F(2,151)=39.46, ****p<0.0001). Both groups exhibited hypersensitivity at WK1 compared to baseline (****p<0.0001). While sham-exposed mice returned to baseline thresholds by WK4, pSNL mice continued to show significant hypersensitivity (****p<0.0001). Solid lines depict the median and dashed lines depict quartiles. Sample sizes are shown in parentheses (Male N, Female N). (N = 64 Male Sham Mice; N = 8 Female Sham Mice; N = 79 Male pSNL Mice; N = 5 Female pSNL Mice).

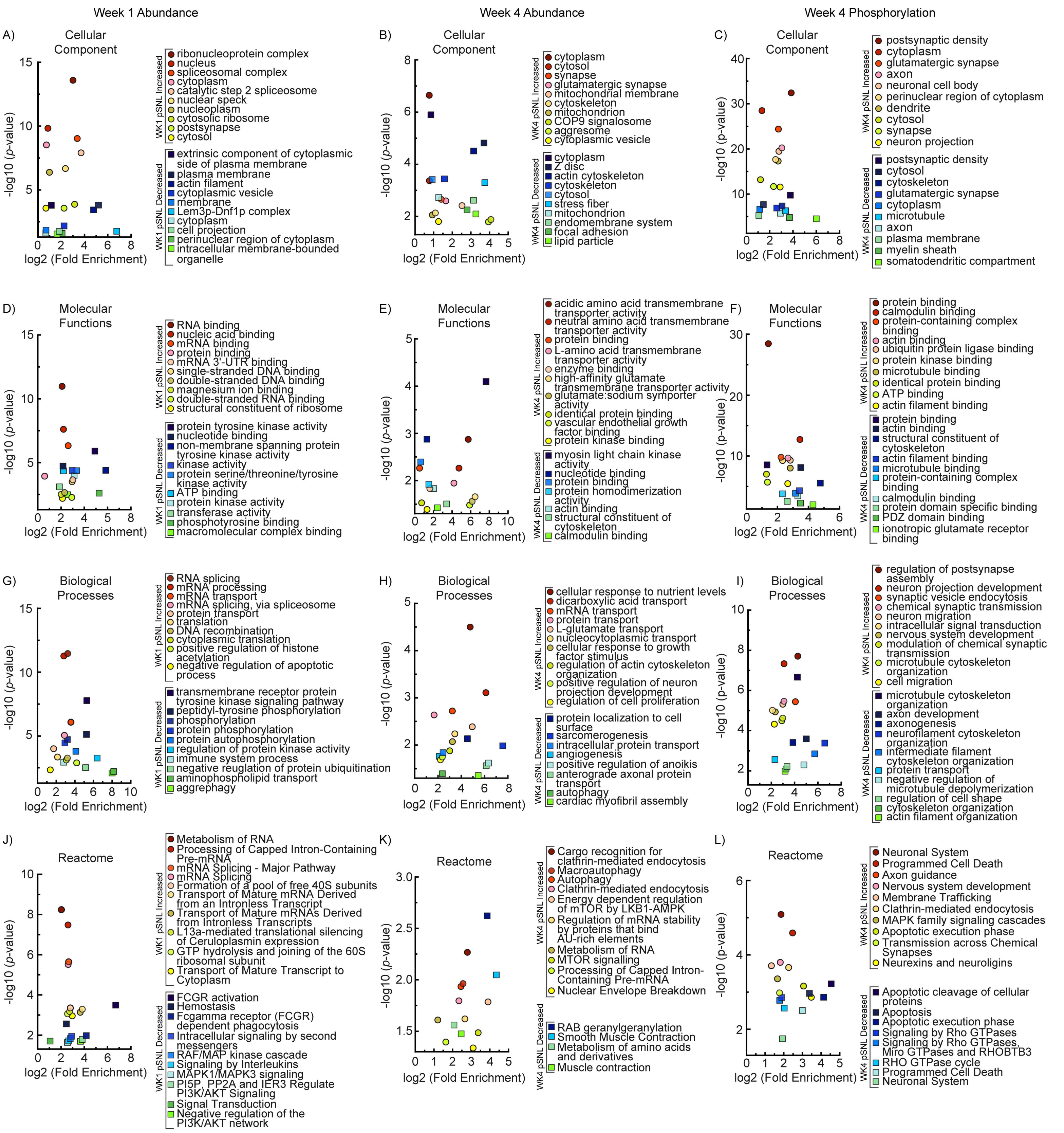

Figure legend is on the next page

Supplementary Figure S2. Broad Proteomic and Phosphoproteomic Enrichment Analysis using DAVID.
(A,D,G,J) Gene Ontology (GO) and Reactome pathway enrichment analysis of DEPs of male WK1 proteome. Enriched cellular components (CC), biological processes (BP), molecular functions (MF), and pathways indicate an early adaptive response involving phosphorylation, RNA binding, Akt signaling, and signal transduction. (N = 3 pooled WK1 Sham samples from male mice; N = 4 pooled WK1 pSNL samples from male mice; 2 bilateral punches/sample).
(B,E,H,K) GO and Reactome pathway enrichment analysis of DEPs of male WK4 proteome. Enriched terms indicate a late maladaptive state involving autophagy, glutamate transport, transporter trafficking, and mTOR signaling. (N = 5 pooled WK4 Sham samples from male mice; N = 5 pooled WK4 pSNL samples from male mice; 2 bilateral punches/sample).
(C,F,I,L) GO and Reactome pathway enrichment analysis of differentially expressed phosphorylated protein at WK4 phosphoproteome, showing significant alterations in proteins related to cellular components like the postsynaptic density, biological processes like neuronal projection development, molecular functions like calmodulin binding, and Reactome pathways like Neuronal System and Programmed Cell Death. (N = 3 pooled WK4 Sham samples from male mice; N = 3 pooled WK4 pSNL samples from male mice; 3 bilateral punches/sample).

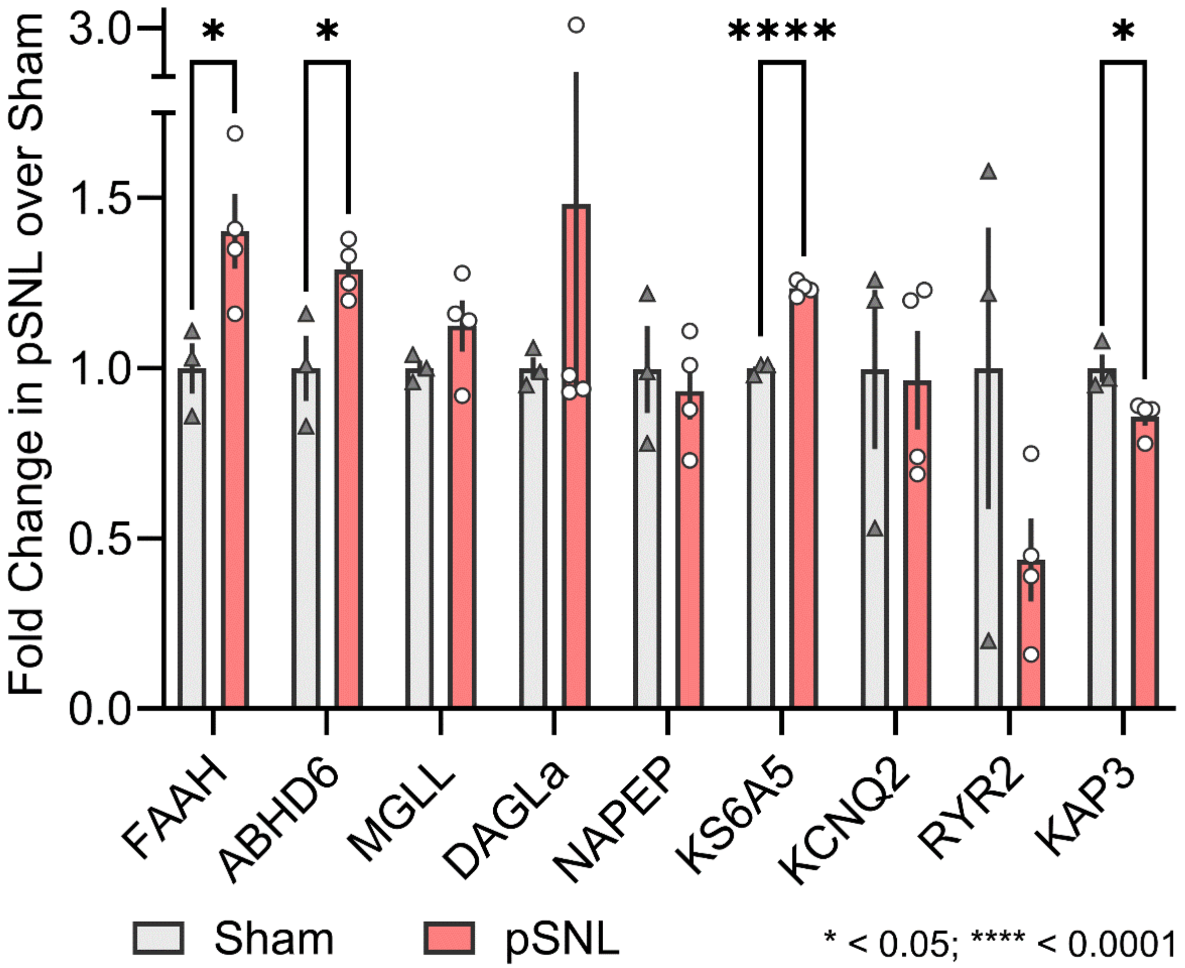

Supplementary Figure S3. Proteomic Analysis of Endocannabinoid-Related Enzymes at Week 1. Bar graph showing the relative abundance of proteins involved in endocannabinoid (eCB) metabolism and synthesis in the VTA at WK1, normalized to sham controls. Significant increases were observed in the degradative enzymes FAAH (*p=0.0306) and ABHD6 (*p=0.0299). Data are expressed as mean ± SEM. (N = 3 pooled WK1 Sham samples from male mice; N = 4 pooled WK1 pSNL samples from male mice; 2 bilateral punches/sample).

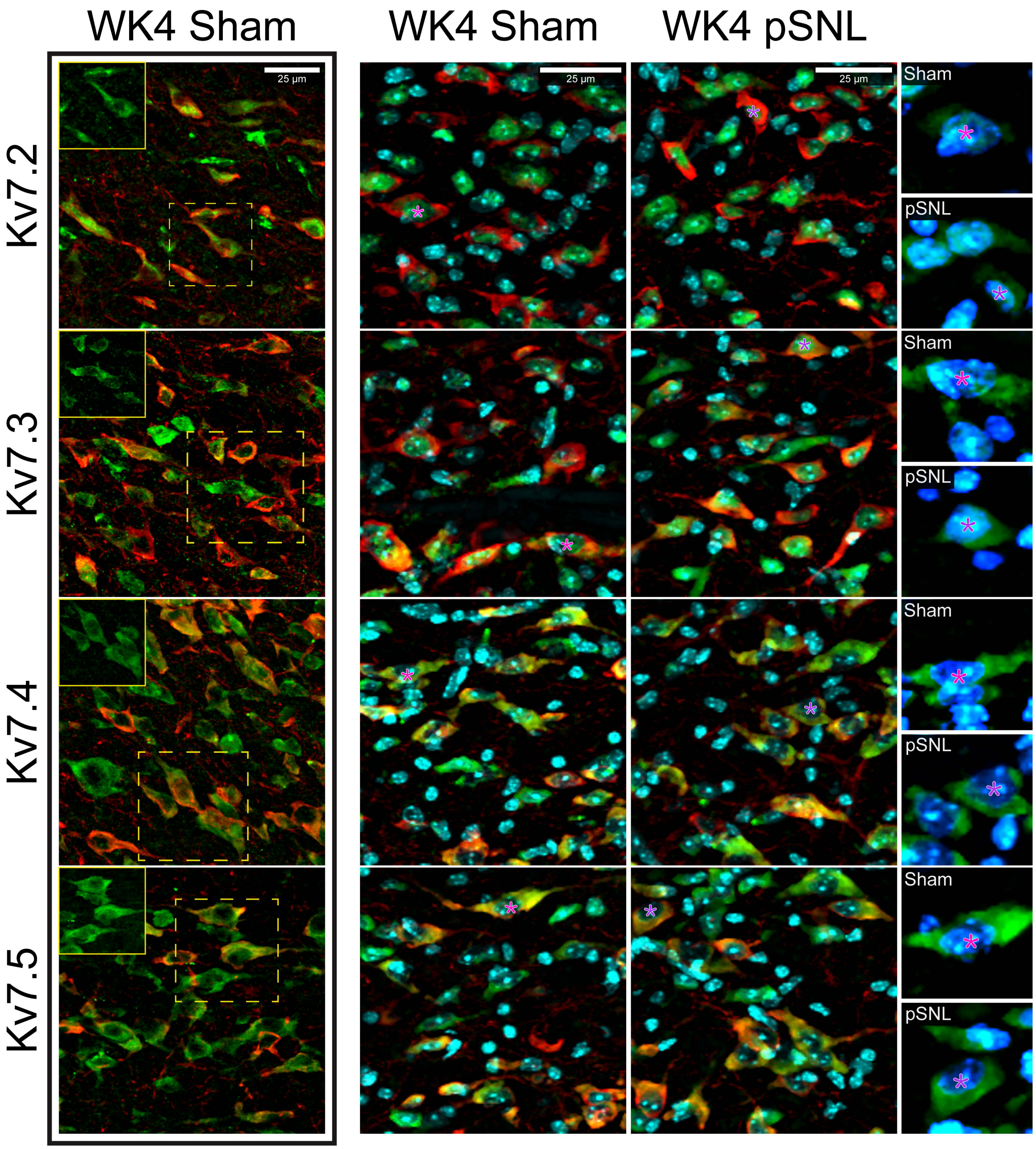

Supplementary Figure S4. Cytosolic Expression of Kv7.2-5 Subunits in VTA Neurons. High-magnification confocal images from the VTA of WK4 male mice (sham and pSNL). Images show co-localization of TH (red), Kv7.2-5 subunits (green), and DAPI (blue), confirming the cytosolic expression pattern of the channels within VTA neurons. Scale bars represent 25 µm.

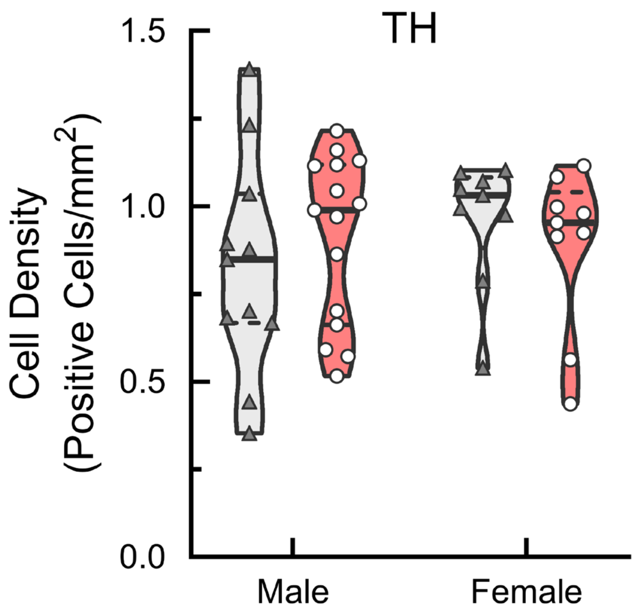

Supplementary Figure S5. VTA TH+ cell density. Violin plots quantifying TH+ cell density in VTA sections co-labeled for TH and Kv7.2 in male and female sham and pSNL mice. A two-way ANOVA was performed on TH+ cell density to evaluate changes in the VTA dopaminergic neuron population. There was no significant Sex × Surgery interaction ($F(1,40) = 1.039$, $p = 0.3143$) and no main effects of Sex ($F(1,40) = 0.4795$, $p = 0.4926$) or Surgery ($F(1,40) = 0.0014$, $p = 0.9706$). (n = 11 male sham VTA slices; n = 15 male pSNL VTA slices; n = 9 female sham VTA slices; n = 9 female pSNL VTA slices; 2–3 VTA slices were analyzed per animal.)
